# Supplementary material for: The latent tuberculosis cascade-of-care among people living with HIV: A systematic review and meta-analysis
Source: PLoS Med. 2021 Sep 7;18(9):e1003703. doi: 10.1371/journal.pmed.1003703 (PMC8439450; doi:10.1371/journal.pmed.1003703)
Supplement: S1 Search Strategy — (DOCX) [file pmed.1003703.s002.docx]

**Search Strategy (Medline Ovid)**

1. exp Tuberculosis/

2. exp Latent Tuberculosis/

3. (Latent TB or latent tuberc* or tuberc* infection or inactive tuber* or LTBI).ti,ab,kw.

4. exp HIV/ or exp HIV Infections/

5. ((Person or People or Patient) adj3 HIV).ti,ab,kw.

6. (HIV or PLHIV* or PLWHIV* or AIDS).ti,ab,kw.

7. exp acquired immunodeficiency syndrome/

8. exp Patient Compliance/

9. exp Medication Adherence/

10. exp "Treatment Adherence and Compliance"/

11. exp Treatment Outcome/

12. exp "Continuity of Patient Care"/

13. (cascade adj2 care).ti,ab,kw.

14. exp "Health Services Needs and Demand"/

15. (complian* or complet* or finaliz* or adher* or contact or yield).ti,ab,kw.

16. exp Mass Screening/

17. (screening or investigation or contact or finding or tuberc* screening).ti,ab,kw.

18. 1 or 2 or 3

19. 4 or 5 or 6 or 7

20. 8 or 9 or 10 or 11 or 12 or 13 or 14 or 15

21. 16 or 17

22. 18 and 19 and 20 and 21

23. limit 22 to yr="2014 -Current

**Search Strategy (LILACS using combination of English, Spanish and Portuguese terms)**

1. ((mh:(tuberculosis)) OR (mh:("latent tuberculosis")) OR (tw:tb) OR (tw: ltbi) OR ((tw: latent OR tw: infect* OR tw: inactive) AND (tw: tuberc* OR tw:tb))) OR ((mh: tuberculose) OR (mh: "tuberculose latente") OR (tw: tb) OR (tw: iltb) OR ((tw: latente OR tw: infec* OR tw”: inativ*) AND (tw: tuberc* OR tw:tb))) OR ((mh: tuberculosis) OR (mh: "Tuberculosis Latente") OR (tw: tb) OR (tw: tbc) OR (tw: itbl) OR ((tw: infecci* OR tw:inactiv* OR tw:latante) AND (tw:tb OR tw:tuberc* OR tw:tbc)))

2. ((mh: hiv) OR (mh: "HIV Infections") OR (mh: "Acquired Immunodeficiency Syndrome") OR (tw: hiv) OR (tw: plhiv*) OR (tw: plwhiv*) OR (tw:aids) OR (((tw: person OR tw: people OR tw: patient) AND tw: hiv)) ) OR ((mh: hiv) OR (mh: "Infecções por HIV") OR (mh: "Síndrome de Imunodeficiência Adquirida") OR (tw: hiv) OR (tw: aids) OR (tw: pvhiv) OR ((tw: pessoa* OR tw: paciente*) AND (tw:hiv))) OR ((mh: vih) OR (mh: "Infecciones por VIH") OR (mh: "Síndrome de Inmunodeficiencia Adquirida") OR (tw: vih) OR (tw: sida) OR (tw: pvvih) OR (tw: pvv) OR ((tw: gente OR tw:persona* OR tw: paciente* OR tw: sujeto) AND (tw: vih)))

3. ((mh: "Patient Compliance") OR (mh: "Medication Adherence") OR (mh: "Treatment Adherence and Compliance") OR (mh: "Treatment Outcome") OR (mh: "Continuity of Patient Care") OR (mh: "Health Services Needs and Demand") OR (tw: complian*) OR (tw: complet*) OR (tw: finaliz*) OR (tw: adher*) OR (tw: contact*) OR (tw: yield) OR (tw: cascade AND tw: care)) OR ((mh: " Cooperação do Paciente") OR (mh: "Adesão à Medicação") OR (mh: "Cooperação e Adesão ao Tratamento") OR (mh: "Resultado do Tratamento") OR (mh: "Continuidade da Assistência ao Paciente") OR (mh: "Necessidades e Demandas de Serviços de Saúde") OR (tw: termin*) OR (tw: adesão) OR (tw: desfecho) OR (tw: fim) OR (tw: comp*) OR (tw: coopera*) OR (tw: cascata AND tw: cuidado)) OR ((mh: "Cooperación del Paciente") OR (mh: "Cumplimiento de la Medicación") OR (mh: "Cumplimiento y Adherencia al Tratamiento") OR (mh: "Resultado del Tratamiento") OR (mh: "Continuidad de la Atención al Paciente") OR (mh: "Necesidades y Demandas de Servicios de Salud") OR (tw: cumplimiento) OR (tw: adherencia) OR (tw: apego) OR (tw: desenlace) OR ((tw: cascada AND (tw: tratamiento OR tw: atención))))

4. (((tw: screening) OR (tw: investigation) OR (mh: "Mass Screening") OR (tw: finding) OR (tw: contact))) OR ((mh: "Programas de Rastreamento") OR (tw: rastre*) OR (tw: investiga*) OR (tw:conta*)) OR ((mh: "Tamizaje Masivo") OR (tw: tamizaje) OR (tw: cribado) OR (tw: investigación ) OR (tw: hallazgo ) OR (tw: contac*))

5. 1 AND 2 AND 3 AND 4
