# Supplementary material for: The latent tuberculosis cascade-of-care among people living with HIV: A systematic review and meta-analysis
Source: PLoS Med. 2021 Sep 7;18(9):e1003703. doi: 10.1371/journal.pmed.1003703 (PMC8439450; doi:10.1371/journal.pmed.1003703)
Supplement: S1 Table — (DOCX) [file pmed.1003703.s003.docx]

# S1 Table. Quality assessment tool used in review for observational studies (adapted from New-Castle Ottawa Scale)

| Risk of bias | Ascertainment of Exposure | Ascertainment of  Outcome | Population Selection |
| --- | --- | --- | --- |
| Low | Secure record | Independent blind assessment | Random selection or consecutive sample – with >80% participation in study |
|  | Structured interview | Record linkage | - |
| Unclear | No description | No description | - |
| High | Written self report | Self report | Random selection or consecutive sample with <80% in the study |
|  |  |  | convenience sample, or sampling method not described |
|  |  |  | Participants were systematically different from non-participants (examples: study participants had more access to tests (TST, CXR) in the facilities where were screening, or, higher socio-economic status, or participants had financial incentives to continue the LTBI investigation |
