# Supplementary material for: The latent tuberculosis cascade-of-care among people living with HIV: A systematic review and meta-analysis
Source: PLoS Med. 2021 Sep 7;18(9):e1003703. doi: 10.1371/journal.pmed.1003703 (PMC8439450; doi:10.1371/journal.pmed.1003703)
Supplement: S2 Table — (DOCX) [file pmed.1003703.s004.docx]

# S2 Table. Quality assessment tool used in review for cluster randomized trials (adapted from Cochrane RoB tool)

| Domains | Question |
| --- | --- |
| Randomization | Was the allocation sequence random? |
|  | Were there baseline imbalances that suggest a problem with the randomization process? |
| Bias arising from the timing of identification and recruitment of individual participants in relation to timing of randomization | Were all the individual participants identified before randomization of clusters (and if the trial specifically recruited patients were, they all recruited before randomization of clusters |
|  | If N/PN/NI to previous question: Is it likely that selection of individual participants was affected by knowledge of the intervention? |
|  | Were there baseline imbalances that suggest differential identification or recruitment of individual participants between arms |
| Bias due to deviations from intended interventions | Were participants aware that they were in a trial |
|  | Were any participants analysed in a group different from the one to which their original cluster was randomized |
|  | If Y/PY/NI: Was there potential for a substantial impact (on the estimated effect of intervention) of analysing participants in the wrong group? |
| Bias due to missing outcome data/measurement of outcome | Were outcome data available for at least 90% of participants within clusters |
|  | Are the proportions of missing outcome data and reasons for missing outcome data similar across intervention groups |
|  | Were outcome assessors blinded? |
