# Supplementary material for: The latent tuberculosis cascade-of-care among people living with HIV: A systematic review and meta-analysis
Source: PLoS Med. 2021 Sep 7;18(9):e1003703. doi: 10.1371/journal.pmed.1003703 (PMC8439450; doi:10.1371/journal.pmed.1003703)
Supplement: S5 Table — (DOCX) [file pmed.1003703.s007.docx]

# S5 Table. Number and clinical characteristics of participants in the studies included in the review

| Study [ref] | N included | Male: Female | Age estimate (mean/median) | N Patients on ARV | CD4 estimate cells/mm3 (mean/median) | LTBI regimen prescribed |
| --- | --- | --- | --- | --- | --- | --- |
| Cowger, Thai (1)^1^ | 789 | 419:370 | Unclear | 0^3^ | Unclear | Isoniazid regimen |
|  | 659 | 330:329 | Unclear | 0^3^ | Unclear | Isoniazid regimen |
| Mueller, Mpala (2) | 1021 | 224:472 | 38.0 | 622 | 433 | Isoniazid regimen |
| Kall, Coyne (3) | 520 | 224:296 | Unclear | 334 | 458 | Mainly Isoniazid regimen (83%) plus 3HR (12%), 2RHZ (5 and 47 (84%) %) |
| Khawcharoenporn, Phetsuksiri (4) | 150 | 79:71 | 40.0 | 113 | Unclear | Isoniazid regimen |
| Aisu, Raviglione (5) | 1524 | Unclear | Unclear | Unclear | Unclear | Isoniazid regimen |
| Diaz, Diez (6) | 1242 | 40:141 | Unclear | 0^3^ | Unclear | Mainly Isoniazid regimen (81%) plus 2RZ (14%), 3HR (4%), 3HRZ (1%) |
| Marks, Taylor (7) | 80 | Unclear | Unclear | Unclear | Unclear | Isoniazid regimen |
| Huerga, Mueller (8) | 897 | 178:372 | 39.0 | 460 | 564 | Isoniazid regimen |
| Doyle, Bissessor (9) | 1378 | 1219:302 | 41.0 | Unclear | 491 | Isoniazid regimen |
| Lee, Lee (10) | 474 | 358:116 | Unclear | 237 | Unclear | Isoniazid regimen |
| Rose, Kitai (11) | 83 | 40:43 | 12.5 | 68 | 827 | Isoniazid regimen |
| Lee, Lin (12) | 774 | 744:28 | 36.8 | 478 | 460 | Isoniazid regimen |
| Pullar, Steinum (13) | 304 | 137:161 | 43.6 | 198 | 410.5 | Mainly 3-4HR |
| Aichelburg, Reiberger (14) | 1525 | 597:249 | 39.6 | 446 | 413 | Unclear |
| Graves, Augusto (15) | 78 | Unclear | Unclear | Unclear | Unclear | Unclear |
| Bourgarit, Baron (16) | 506 | 307:108 | 38.9 | 0^3^ | 483 | Unclear |
| Xin, Li (17) | 195 | 195:0 | 34.0 | 156 | 390 | Isoniazid regimen |
| Pascopella, Franks (18) | 600 | 419:181 | 46.0 | 573 | 182 | Isoniazid regimen |
| Leung, Chan (19) | 110 | 96:14 | 42.7 | 930 | 414 | Isoniazid regimen |
| Al-Darraji, Kamarulzaman (20) | 154 | 124: 14 | 37.5 | Unclear | 384 | Unclear |
| Reaves, Shah (21) | 2772 | 2037:735 | Unclear | 2388 | Unclear | Unclear |
| Hirsch-Moverman, Cronin (22) | 71 | 43:28 | Unclear | Unclear | Unclear | Unclear |
| Fox-Lewis, Brima (23) | 165 | Unclear | Unclear |  | Unclear | Unclear |
| Sester, Van Leth (24) | 768 | 566:202 | 40.8 | Unclear | Unclear | Unclear |
| Sun, Hsueh (25) | 617 | 589:19 | 45.0 | 550 | 497.8 | Not applicable |
| Zhang, Liu (26) | 50 | 24:26 | Unclear | 50 | 305 | Not applicable |
| Yang, Chan (27) | 1001 | 796:113 | 34.8 | 369 | 447 | Isoniazid regimen |
| Shin, Chang (28) | 15346 | 13180:1849 | Unclear | Unclear | Unclear | Isoniazid regimen |
| Cheng, Hirji (29) | 129 | 88:33 | 43.0 | 49 | 135 | Unclear |
| Goletti, Navarra (30) | 774 | 557:154 | 38.0 | 0^3^ | 319 | Unclear |
| Stein, Zalwango (31) | 197 | Unclear | Unclear | Unclear | Unclear | Isoniazid regimen |
| Meijerink, Wisaksana (32) | 524 | 351:173 | 31.0 | 524 | 373 | Isoniazid regimen |
| Adams, Howe (33) | 372 | 253:119 | Unclear | 330 | Unclear | Unclear |
| Brassard, Bruneau (34) | 60 | Unclear | Unclear | Unclear | Unclear | Isoniazid regimen |
| Golub, Cohn (35) | 2077 | 1216:738 | Unclear | 1061 | Unclear | Isoniazid regimen |
| Lobato, Leary (36) | 4435 | Unclear | Unclear | Unclear | Unclear | Isoniazid regimen |
| Martinez-Pino, Sambeat (37) | 7977 | 6007:1970 | 37.7 | 644 | 415 | Isoniazid regimen |
| Elzi, Schlegel (38) | 6160 | 4189:1971 | 36.0 | 2526 | 326 | Mainly Isoniazid regimen (77%), plus other scarce regimens: Rfb+Z (16%), RZ (2%), Rfb (4%), R (1.5%) |
| Wong, Leung (39) | 2079 | 1752:327 | 35.0 | 1742 | 312 | Isoniazid regimen |
| Capocci, Sewell (40) | 982 | 693:289 | 46.0 | 907 | 643 | 3HR, Isoniazid regimen |
| Sandhu, Taylor (41) | 206 | 134:72 | 42.1 | Unclear | 352 | Unclear |
| Baker, Peterson (42) | 129 | Unclear | Unclear | Unclear | Unclear | Isoniazid regimen |
| Froberg, Jansson (43) | 69 | Unclear | Unclear | Unclear | Unclear | 3HR, 4R and Isoniazid regimen |
| Santos, Garcia (44) | 872 | 413:277 | Unclear | Unclear | Unclear | Isoniazid regimen |
| Picone, Freitas (45) | 3260 | 168:70 | 42.0 | 211 | Unclear | Isoniazid regimen |
| Kussen, Dalla-Costa (46) | 154 | 67:73 | Unclear | 121 | Unclear | Isoniazid regimen |
| Monteiro, Guariente (47) | 220 | 131:89 | Unclear | 148 | Unclear | Isoniazid regimen |
| Aquino, Moura (48) | 2341 | 133:99 | Unclear | 187 | Unclear | Isoniazid regimen |
| Souza, Hökerberg (49) | 138 | 90:48 | 41.0 | 102 | Unclear | Isoniazid regimen |
| Trinh, Han (50) | 1281 | 861:420 | 32.0 | 199 | 298 | Isoniazid regimen |
| van Griensven, Choun (51) | 875 | 157:288 | 35.0 | 306 | 354 | Isoniazid regimen |
| Little, Khundi (52) | 1359 | 324:650 | 33.0 | 152 | Unclear | Isoniazid regimen |
| Benzekri, Sambou (53) | 198 | 74:124 | Unclear | 168 | 185 | Isoniazid regimen |
| Van Ginderdeuren, Bassett (54) | 1395 | 298:627 | 39.0 | 814 | 423 | Isoniazid regimen |
| Deery, Hanrahan (55) | 27 | Unclear | Unclear | 3 | 389 | Isoniazid regimen |
| Sah, Sahu (56) | 572 | 314:258 | Unclear | 521 | Unclear | Isoniazid regimen |
| Tiam, Machekano (57) | 160 | 0:160 | 26.0 | 58 | Unclear | Isoniazid regimen |
| Costenaro, Massavon (58) | 899 | 441:448 | 10.7 | 572 | Unclear | Isoniazid regimen |
| Peters, Heunis (59) | 71 | 0:71 | Unclear | 14 | Unclear | Isoniazid regimen |
| Kufa, Fielding (60)2 | 1540 | 608:932 | 34.2 | 597 | 200 | Isoniazid regimen |
|  | 1484 | 530:954 | 33.7 | 626 | 210 | Isoniazid regimen |
| Carmone, Rodriguez (61) | 532 | Unclear | Unclear | Unclear | Unclear | Isoniazid regimen |
| Adams, Mahlalela (62) | 908 | 307:601 | 38.0 | 868 | Unclear | Isoniazid regimen |
| Shayo, Moshiro (63) | 1283 | 289:994 | Unclear | 982 | Unclear | Isoniazid regimen |
| Roscoe, Lockhart (64) | 861 | 314:485 | 33.0 | 640 | Unclear | Isoniazid regimen |
| Igbokwe, Abugu (65) | 993 | 292:701 | Unclear | 993 | Unclear | Isoniazid regimen |
| Hunter, Kyesi (66) | 66 | 23:43 | 11.0 | 66 | Unclear | Isoniazid regimen |
| Adepoju, Ogbudebe (67) | 7791 | 740:394 | 1101 | 1101 | Unclear | Isoniazid regimen |
| Karanja, Kingwara (68) | 4708 | 1549:3159 | Unclear | 4525 | Unclear | Isoniazid regimen |
| Melgar, Nichols (69) | 34,605,241 | Unclear | Unclear | Unclear | Unclear | Unclear |

References

1. Cowger TL, Thai LH, Duong BD, Danyuttapolchai J, Kittimunkong S, Nhung NV, et al. Programmatic evaluation of an algorithm for intensified tuberculosis case finding and isoniazid preventive therapy for people living with HIV in Thailand and Vietnam. Journal of Acquired Immune Deficiency Syndromes. 2017;76(5):512-21. PubMed PMID: 621681989.

2. Mueller Y, Mpala Q, Kerschberger B, Rusch B, McHunu G, Mazibuko S, et al. Adherence, tolerability, and outcome after 36 months of isoniazid-preventive therapy in 2 rural clinics of Swaziland. Medicine (United States). 96. PubMed PMID: 618161560.

3. Kall MM, Coyne KM, Garrett NJ, Boyd AE, Ashcroft AT, Reeves I, et al. Latent and subclinical tuberculosis in HIV infected patients: a cross-sectional study. BMC Infect Dis. 12:107. PubMed PMID: 22558946.

4. Khawcharoenporn T, Phetsuksiri B, Rudeeaneksin J, Srisungngam S, Apisarnthanarak A. QuantiFERON-TB Gold In-Tube Test for Tuberculosis Prevention in HIV-Infected Patients. Japanese Journal of Infectious Diseases. 2017;70:502-6. PubMed PMID: r.

5. Aisu T, Raviglione MC, van Praag E, Eriki P, Narain JP, Barugahare L, et al. Preventive chemotherapy for HIV-associated tuberculosis in Uganda: an operational assessment at a voluntary counselling and testing centre. Aids. 9(3):267-73. PubMed PMID: 7755915.

6. Diaz A, Diez M, Bleda MJ, Aldamiz M, Camafort M, Camino X, et al. Eligibility for and outcome of treatment of latent tuberculosis infection in a cohort of HIV-infected people in Spain. BMC Infect Dis. 10:267. PubMed PMID: 20840743.

7. Marks SM, Taylor Z, Qualls NL, Shrestha-Kuwahara RJ, Wilce MA, Nguyen CH. Outcomes of contact investigations of infectious tuberculosis patients. Am J Respir Crit Care Med. 162(6):2033-8. PubMed PMID: 11112109.

8. Huerga H, Mueller Y, Ferlazzo G, Mpala Q, Bevilacqua P, Vasquez B, et al. Implementation and Operational Research: Feasibility of Using Tuberculin Skin Test Screening for Initiation of 36-Month Isoniazid Preventive Therapy in HIV-Infected Patients in Resource-Constrained Settings. J Acquir Immune Defic Syndr. 2016;71(4):e89-95. Epub 2016/02/26. doi: 10.1097/qai.0000000000000895. PubMed PMID: 26910386.

9. Doyle JS, Bissessor M, Denholm JT, Ryan N, Fairley CK, Leslie DE. Latent tuberculosis screening using interferon-gamma release assays in an australian HIV-infected cohort: Is routine testing worthwhile? Journal of Acquired Immune Deficiency Syndromes. 66(1):48-54. PubMed PMID: 52976529.

10. Lee S, Lee JE, Kang JS, Lee SO, Lee SH. Long-term performance of the IGRA to predict and prevent active tuberculosis development in HIV-infected patients. International Journal of Tuberculosis and Lung Disease. 23(4):422-7. PubMed PMID: 2001979764.

11. Rose W, Kitai I, Kakkar F, Read SE, Behr MA, Bitnun A. Quantiferon Gold-in-tube assay for TB screening in HIV infected children: Influence of quantitative values. BMC Infectious Diseases. 14(516). PubMed PMID: 600063820.

12. Lee SS, Lin HH, Tsai HC, Su IJ, Yang CH, Sun HY, et al. A Clinical Algorithm to Identify HIV Patients at High Risk for Incident Active Tuberculosis: A Prospective 5-Year Cohort Study. PLoS ONE. 2015;10(8):e0135801. PubMed PMID: 26280669.

13. Pullar ND, Steinum H, Bruun JN, Dyrhol-Riise AM. HIV patients with latent tuberculosis living in a low-endemic country do not develop active disease during a 2 year follow-up; a Norwegian prospective multicenter study. BMC Infect Dis. 14:667. PubMed PMID: 25515915.

14. Aichelburg MC, Reiberger T, Breitenecker F, orfer M, Makristathis A, Rieger A. Reversion and conversion of interferon-gamma release assay results in HIV-1-infected individuals. J Infect Dis. 209(5):729-33. PubMed PMID: 23911707.

15. Graves SK, Augusto O, Viegas SO, Lederer P, David C, Lee K, et al. Tuberculosis infection risk, preventive therapy care cascade and incidence of tuberculosis disease in healthcare workers at Maputo Central Hospital. BMC Infectious Diseases. 19(1):346. PubMed PMID: 31023260.

16. Bourgarit A, Baron G, Breton G, Tattevin P, Katlama C, Allavena C, et al. Latent tuberculosis infection screening and 2-year outcome in antiretroviral-naive HIV-infected patients in a low-prevalence country. Annals of the American Thoracic Society. 12(8):1138-45. PubMed PMID: 606143998.

17. Xin HN, Li XW, Zhang L, Li Z, Zhang HR, Yang Y, et al. Tuberculosis infection testing in HIV-positive men who have sex with men from Xi'an China. Epidemiology and Infection. 145(3):498-502. PubMed PMID: 613340710.

18. Pascopella L, Franks J, Marks SM, Salcedo K, Schmitz K, Colson PW, et al. Opportunities for tuberculosis diagnosis and prevention among persons living with HIV: A cross-sectional study of policies and practices at four large Ryan White program-funded HIV clinics. PLoS ONE. 9. PubMed PMID: 373528878.

19. Leung CC, Chan K, Yam WC, Lee MP, Chan CK, Wong KH, et al. Poor agreement between diagnostic tests for latent tuberculosis infection among HIV-infected persons in Hong Kong. Respirology. 2016. PubMed PMID: 610175911.

20. Al-Darraji HA, Kamarulzaman A, Altice FL. Latent tuberculosis infection in a Malaysian prison: implications for a comprehensive integrated control program in prisons. BMC public health. 2014;14:22. PubMed PMID: 605670147.

21. Reaves EJ, Shah NS, France AM, Morris SB, Kammerer S, Skarbinski J, et al. Latent tuberculous infection testingamongHIV-infected persons in clinical care, United States, 2010-2012. International Journal of Tuberculosis and Lung Disease. 21(10):1118-26. PubMed PMID: 618346045.

22. Hirsch-Moverman Y, Cronin WA, Chen B, Moran JA, Munk E, Reichler MR. HIV counseling and testing in tuberculosis contact investigations in the United States and Canada. International Journal of Tuberculosis and Lung Disease. 19(8):943-53. PubMed PMID: 605446742.

23. Fox-Lewis A, Brima N, Muniina P, Grant AD, Edwards SG, Miller RF, et al. Tuberculosis screening in patients with HIV: An audit against UK national guidelines to assess current practice and the effectiveness of an electronic tuberculosis-screening prompt. International Journal of STD and AIDS. 27(10):901-5. PubMed PMID: 611908996.

24. Sester M, Van Leth F, Bruchfeld J, Bumbacea D, Cirillo DM, Dilektasli AG, et al. Risk assessment of tuberculosis in immunocompromised patients: A TBNET study. American Journal of Respiratory and Critical Care Medicine. 190(10):1168-76. PubMed PMID: 604384400.

25. Sun HY, Hsueh PR, Liu WC, Su YC, Chang SY, Hung CC, et al. Risk of Active Tuberculosis in HIV-Infected Patients in Taiwan with Free Access to HIV Care and a Positive T-Spot.TB Test. PLoS ONE. 2015;10(5):e0125260. PubMed PMID: 25938227.

26. Zhang LF, Liu XQ, Zuo LY, Li TS, Deng GH, Wang AX. Longitudinal observation of an interferon gamma-released assay (T-SPOT.TB) for Mycobacterium tuberculosis infection in AIDS patients on highly active antiretroviral therapy. Chin Med J (Engl). 123(9):1117-21. PubMed PMID: 20529548.

27. Yang CH, Chan PC, Liao ST, Cheng SH, Wong WW, Huang LM, et al. Strategy to better select HIV-infected individuals for latent TB treatment in BCG-vaccinated population. PLoS ONE. 2013;8(8):e73069. PubMed PMID: 24015285.

28. Shin SS, Chang AH, Ghosh JK, Dubé MP, Bolan R, Yang OO, et al. Isoniazid therapy for Mycobacterium tuberculosis infection in HIV clinics, Los Angeles, California. Int J Tuberc Lung Dis. 2016;20(7):961-6. Epub 2016/06/12. doi: 10.5588/ijtld.15.0988. PubMed PMID: 27287651; PubMed Central PMCID: PMCPMC4905690.

29. Cheng MP, Hirji A, Roth DZ, Cook VJ, Lima VD, Montaner JS, et al. Tuberculosis in HIV-infected persons in British Columbia during the HAART era. Canadian journal of public health = Revue canadienne de sante publique. 105(4):e258-e62. PubMed PMID: 603675458.

30. Goletti D, Navarra A, Petruccioli E, Cimaglia C, Compagno M, Cuzzi G, et al. Latent tuberculosis infection screening in persons newly-diagnosed with HIV infection in Italy: a multicentre study promoted by the Italian Society of Infectious and Tropical Diseases. International journal of infectious diseases : IJID : official publication of the International Society for Infectious Diseases. 2019;27. PubMed PMID: 630481985.

31. Stein CM, Zalwango S, Malone LL, Thiel B, Mupere E, Nsereko M, et al. Resistance and Susceptibility to Mycobacterium tuberculosis Infection and Disease in Tuberculosis Households in Kampala, Uganda. American Journal of Epidemiology. 187(7):1477-89. PubMed PMID: 623483838.

32. Meijerink H, Wisaksana R, Lestari M, Meilana I, Chaidir L, Van Der Ven AJAM, et al. Active and latent tuberculosis among HIV-positive injecting drug users in Indonesia. Journal of the International AIDS Society. 18. PubMed PMID: 602273136.

33. Adams JW, Howe CJ, Andrews AC, Allen SL, Vinnard C. Tuberculosis screening among HIV-infected patients: tuberculin skin test vs. interferon-gamma release assay. AIDS Care - Psychological and Socio-Medical Aspects of AIDS/HIV. 29(12):1504-9. PubMed PMID: 616101560.

34. Brassard P, Bruneau J, Schwartzman K, Senecal M, Menzies D. Yield of tuberculin screening among injection drug users. Int J Tuberc Lung Dis. 8(8):988-93. PubMed PMID: 15305482.

35. Golub JE, Cohn S, Saraceni V, Cavalcante SC, Pacheco AG, Moulton LH, et al. Long-term protection from isoniazid preventive therapy for tuberculosis in HIV-infected patients in a medium-burden tuberculosis setting: the TB/HIV in Rio (THRio) study. Clin Infect Dis. 60(4):639-45. PubMed PMID: 25365974.

36. Lobato MN, Leary LS, Simone PM. Treatment for latent TB in correctional facilities: a challenge for TB elimination. Am J Prev Med. 24(3):249-53. PubMed PMID: 12657343.

37. Martinez-Pino I, Sambeat MA, Lacalle-Remigio JR, Domingo P. Incidence of tuberculosis in HIV-infected patients in Spain: the impact of treatment for LTBI. Int J Tuberc Lung Dis. 17(12):1545-51. PubMed PMID: 24200266.

38. Elzi L, Schlegel M, Weber R, Hirschel B, Cavassini M, Schmid P, et al. Reducing tuberculosis incidence by tuberculin skin testing, preventive treatment, and antiretroviral therapy in an area of low tuberculosis transmission. Clin Infect Dis. 44(1):94-102. PubMed PMID: 17143823.

39. Wong NS, Leung CC, Chan KCW, Chan WK, Lin AWC, Lee SS. A longitudinal study on latent TB infection screening and its association with TB incidence in HIV patients. Sci Rep. 9. PubMed PMID: WOS:000475292700015.

40. Capocci SJ, Sewell J, Smith C, Cropley I, Bhagani S, Solamalai A, et al. Cost effectiveness of testing HIV infected individuals for TB in a low TB/HIV setting. J Infect Dis. 2020;81(2):289-96.

41. Sandhu P, Taylor C, Miller RF, Post FA. Implementation of routine interferon-gamma release assay testing in a South London HIV cohort. Int J STD AIDS. 31(3):264-7.

42. Baker BJ, Peterson B, Mohanlall J, Singh S, Hicks C, Jacobs R, et al. Scale-up of collaborative TB/HIV activities in Guyana. Rev Panam Salud Publica. 2017;41:e6-e.

43. Froberg G, Jansson L, Nyberg K, Obasi B, Westling K, Berggren I, et al. Screening and treatment of tuberculosis among pregnant women in Stockholm, Sweden, 2016-2017. Eur Respir J. 2020;55(3):03.

44. Santos DTd, Garcia MC, Costa AANFd, Pieri FM, Meier DAP, Albanese SPR, et al. Infecção latente por tuberculose entre pessoas com HIV/AIDS, fatores associados e progressão para doença ativa em município no Sul do Brasil. Cad Saude Publica. 2017;33(8):e00050916-e.

45. Picone CM, Freitas AC, Gutierrez EB, Avelino-Silva VI. Access and adherence to isoniazid preventive therapy and occurrence of active TB in a cohort of people living with HIV: a retrospective cohort study in Sao Paulo, Brazil. Rev Inst Med Trop Sao Paulo. 2020;62:e8.

46. Kussen GMB, Dalla-Costa LM, Rossoni A, Raboni SM. Interferon-gamma release assay versus tuberculin skin test for latent tuberculosis infection among HIV patients in Brazil. Braz J Infect Dis. 2016;20(1):69-75.

47. Monteiro ATA, Guariente MHDdM, Costa AANFd, Santos DTd, Alves E, Dessunti EM. Prova tuberculínica: o controle da tuberculose em pacientes infectados pelo vírus da imunodeficiência humana (HIV). Semin Ciênc Biol Saude. 2015;36(1):179-88.

48. Aquino DSd, Moura LCRV, Maruza M, Silva APd, Ximenes RAdA, Lacerda HR, et al. Factors associated with treatment for latent tuberculosis in persons living with HIV/AIDS. Cad Saude Publica. 2015;31(12):2505-13.

49. Souza CTVd, Hökerberg YHM, Pacheco S, Bedoya rJ, Rolla VC, Passos SRL. Effectiveness and safety of isoniazid chemoprophylaxis for HIV-1 infected patients from Rio de Janeiro. Mem Inst Oswaldo Cruz. 2009;104(3):462-7.

50. Trinh TT, Han DT, Bloss E, Le TH, Vu TT, Mai AH, et al. Implementation and evaluation of an isoniazid preventive therapy pilot program among hiv-infected patients in vietnam, 2008-2010. Transactions of the Royal Society of Tropical Medicine and Hygiene. 109(10):653-9. PubMed PMID: 606407680.

51. van Griensven J, Choun K, Chim B, Thai S, Lorent N, Lynen L. Implementation of isoniazid preventive therapy in an HIV clinic in Cambodia: high rates of discontinuation when combined with antiretroviral therapy. Tropical Medicine & International Health. 20(12):1823-31. PubMed PMID: 26426387.

52. Little KM, Khundi M, Barnes GL, Ngwira LG, Nkhoma A, Makombe S, et al. Predictors of isoniazid preventive therapy completion among adults newly diagnosed with HIV in rural Malawi. International Journal of Tuberculosis and Lung Disease. 22(4):371-7. PubMed PMID: 621576115.

53. Benzekri NA, Sambou JF, Ndong S, Tamba IT, Faye D, Diallo MB, et al. Prevalence, predictors, and management of advanced HIV disease among individuals initiating ART in Senegal, West Africa. BMC Infectious Diseases. 19(261). PubMed PMID: 626772163.

54. Van Ginderdeuren E, Bassett J, Hanrahan C, Mutunga L, Van Rie A. Health system barriers to implementation of TB preventive strategies in South African primary care facilities. PLoS ONE. 14.

55. Deery CB, Hanrahan CF, Selibas K, Bassett J, Sanne I, Van Rie A. A home tracing program for contacts of people with tuberculosis or HIV and patients lost to care. International Journal of Tuberculosis and Lung Disease. 18(5):534-40+i. PubMed PMID: 372973633.

56. Sah SK, Sahu SK, Lamichhane B, Bhatta GK, Bh, ari KB, et al. Dotting the Three I's for collaborative TB-HIV activities: Evaluation of a pilot programme in Kathmandu, Nepal. Public Health Action. 6(3):169-75. PubMed PMID: 612815398.

57. Tiam A, Machekano R, Gounder CR, Maama-Maime LBM, Ntene-Sealiete K, Sahu M, et al. Preventing tuberculosis among HIV-infected pregnant women in lesotho: The case for rolling out active case finding and isoniazid preventive therapy. Journal of Acquired Immune Deficiency Syndromes. 67(1):e5-e11. PubMed PMID: 373763298.

58. Costenaro P, Massavon W, Lundin R, Nabachwa SM, Fregonese F, Morelli E, et al. Implementation and Operational Research: Implementation of the WHO 2011 Recommendations for Isoniazid Preventive Therapy (IPT) in Children Living With HIV/AIDS: A Ugandan Experience. Journal of Acquired Immune Deficiency Syndromes: JAIDS. 71(1):e1-8. PubMed PMID: 26761275.

59. Peters JA, Heunis C, Kigozi G, Osoba T, Van Der Walt M. Integration of TB-HIV services at an anc facility in frances baard district, northern cape, south africa. Public Health Action. 2015;5(1):30-5. PubMed PMID: 603240996.

60. Kufa T, Fielding KL, Hippner P, Kielmann K, Vassall A, Churchyard GJ, et al. An intervention to optimise the delivery of integrated tuberculosis and HIV services at primary care clinics: results of the MERGE cluster randomised trial. Contemporary Clinical Trials. 72:43-52. PubMed PMID: 2000984591.

61. Carmone A, Rodriguez CA, Frank TD, Kiromat M, Bongi PW, Kuno RG, et al. Increasing isoniazid preventive therapy uptake in an HIV program in rural Papua New Guinea. Public Health Action. 7(3):193-8. PubMed PMID: 618478490.

62. Adams LV, Mahlalela N, Talbot EA, Pasipamire M, Ginindza S, Calnan M, et al. High completion rates of isoniazid preventive therapy among persons living with HIV in Swaziland. International Journal of Tuberculosis & Lung Disease. 21(10):1127-32. PubMed PMID: 28911356.

63. Shayo GA, Moshiro C, Aboud S, Bakari M, Mugusi FM. Acceptability and adherence to Isoniazid preventive therapy in HIV-infected patients clinically screened for latent tuberculosis in Dar es Salaam, Tanzania. BMC Infectious Diseases. 15(368). PubMed PMID: 605749328.

64. Roscoe C, Lockhart C, de Klerk M, Baughman A, Agolory S, Gawanab M, et al. Evaluation of the uptake of tuberculosis preventative therapy for people living with HIV in Namibia: a multiple methods analysis. BMC Public Health. 20(1):1838.

65. Igbokwe CC, Abugu LI, Aji JO. Assessment of tuberculosis intensified case finding and isoniazid preventive therapy for people living with hiv in enugu state, nigeria. Afr J Biomed Res. 23(3):367-73.

66. Hunter OF, Kyesi F, Ahluwalia AK, Daffe ZN, Munseri P, von Reyn CF, et al. Successful implementation of isoniazid preventive therapy at a pediatric HIV clinic in Tanzania. BMC Infect Dis. 2020;20(1):738.

67. Adepoju A, Ogbudebe C, Adejumo O, Okolie J, Inegbeboh J. Implementation of isoniazid preventive therapy among people living with HIV in Northwestern Nigeria: Completion rate and predictive factors. J Glob Infect Dis. 12(2):105-11.

68. Karanja M, Kingwara L, Owiti P, Kirui E, Ngari F, Kiplimo R, et al. Outcomes of isoniazid preventive therapy among people living with HIV in Kenya: A retrospective study of routine health care data. PLoS ONE. 2020;15(12):e0234588.

69. Melgar M, Nichols C, Cavanaugh JS, Kirking HL, Surie D, Date A, et al. Tuberculosis Preventive Treatment Scale-Up Among Antiretroviral Therapy Patients - 16 Countries Supported by the U.S. President's Emergency Plan for AIDS Relief, 2017-2019. MMWR Morb Mortal Wkly Rep. 2020;69(12):329-34.
