# Supplementary material for: The latent tuberculosis cascade-of-care among people living with HIV: A systematic review and meta-analysis
Source: PLoS Med. 2021 Sep 7;18(9):e1003703. doi: 10.1371/journal.pmed.1003703 (PMC8439450; doi:10.1371/journal.pmed.1003703)
Supplement: S6 Table — (DOCX) [file pmed.1003703.s008.docx]

# S6 Table. Number of participants in each step of the cascade-of-care among studies that did not use LTBI tests.

| Study [ref] | Used LTBI test | Total identified (eligible for testing & treatment) | Medical Evaluation | Recommended LTBI treatment | Started LTBI treatment | Completed LTBI treatment |
| --- | --- | --- | --- | --- | --- | --- |
| Reported all steps of the cascade of care (all 4 proportions calculated) | | | | | | |
| Trinh, Han (1) | No | 1281 | 520 | 426 | 416 | 382 |
| van Griensven, Choun (2) | No | 875 | 875 | 631 | 445 | 348 |
| Little, Khundi (3) | No | 1359 | 1106 | 1011 | 1010 | 732 |
| Cowger, Thai (4) | No | 789 | 789 | 426 | 245 | 160 |
| Benzekri, Sambou (5) | No | 303 | 166 | 73 | 1 | 0 |
| Roscoe, Lockhart (6) | No | 825 | 679 | 646 | 294 | 139 |
| Reported all steps up to starting LTBI treatment (proportions 1 through 3 calculated) | | | | | | |
| Van Ginderdeuren, Bassett (7) | No | 1395 | 1377 | 1178 | 329 |  |
| Deery, Hanrahan (8) | No | 27 | 15 | 15 | 3 |  |
| Reported the early steps of the cascade (proportion 1 and 2 calculated) | | | | | | |
| Igbokwe, Abugu (9) | No | 993 | 981 | 608 |  |  |
| Reported all steps up to med evaluation and the latest steps of the cascade (proportions 1 & 4) | | | | | | |
| Sah, Sahu (10) | No | 572 | 561 |  | 157 | 136 |
| Tiam, Machekano (11) | No | 160 | 158 |  | 124 | 80 |
| Costenaro, Massavon (12) | No | 899 | 529 |  | 280 | 259 |
| Reported the early steps of the cascade (proportion 1 calculated) | | | | | | |
| Peters, Heunis (13) | No | 71 | 30 |  | 11 |  |
| Kufa, Fielding (14) Cohort 1 | No | 1999 | 1540 |  | 503 |  |
| Kufa, Fielding (14) Cohort 2 | No | 2004 | 1480 |  | 552 |  |
| Missed the first and last steps of the cascade, (proportions 2 and 3 calculated) | | | | | | |
| Carmone, Rodriguez (15) | No |  | 532 | 450 | 450 |  |
| Missed the first and second steps (proportion 3 and 4 calculated) | | | | | | |
| Hunter, Kyesi (16) | No |  |  | 66 | 66 | 49 |
| Only reported the last steps of the cascade (proportion 4 calculated) | | | | | | |
| Adams, Mahlalela (17) | No |  |  |  | 908 | 812 |
| Shayo, Moshiro (18) | No |  |  |  | 1283 | 1255 |
| Adepoju, Ogbudebe (19) |  | 7791 |  |  | 1134 | 454 |
| Karanja, Kingwara (20) |  |  |  |  | 4708 | 3712 |

References

1. Trinh TT, Han DT, Bloss E, Le TH, Vu TT, Mai AH, et al. Implementation and evaluation of an isoniazid preventive therapy pilot program among hiv-infected patients in vietnam, 2008-2010. Transactions of the Royal Society of Tropical Medicine and Hygiene. 109(10):653-9. PubMed PMID: 606407680.

2. van Griensven J, Choun K, Chim B, Thai S, Lorent N, Lynen L. Implementation of isoniazid preventive therapy in an HIV clinic in Cambodia: high rates of discontinuation when combined with antiretroviral therapy. Tropical Medicine & International Health. 20(12):1823-31. PubMed PMID: 26426387.

3. Little KM, Khundi M, Barnes GL, Ngwira LG, Nkhoma A, Makombe S, et al. Predictors of isoniazid preventive therapy completion among adults newly diagnosed with HIV in rural Malawi. International Journal of Tuberculosis and Lung Disease. 22(4):371-7. PubMed PMID: 621576115.

4. Cowger TL, Thai LH, Duong BD, Danyuttapolchai J, Kittimunkong S, Nhung NV, et al. Programmatic evaluation of an algorithm for intensified tuberculosis case finding and isoniazid preventive therapy for people living with HIV in Thailand and Vietnam. Journal of Acquired Immune Deficiency Syndromes. 2017;76(5):512-21. PubMed PMID: 621681989.

5. Benzekri NA, Sambou JF, Ndong S, Tamba IT, Faye D, Diallo MB, et al. Prevalence, predictors, and management of advanced HIV disease among individuals initiating ART in Senegal, West Africa. BMC Infectious Diseases. 19(261). PubMed PMID: 626772163.

6. Roscoe C, Lockhart C, de Klerk M, Baughman A, Agolory S, Gawanab M, et al. Evaluation of the uptake of tuberculosis preventative therapy for people living with HIV in Namibia: a multiple methods analysis. BMC Public Health. 20(1):1838.

7. Van Ginderdeuren E, Bassett J, Hanrahan C, Mutunga L, Van Rie A. Health system barriers to implementation of TB preventive strategies in South African primary care facilities. PLoS ONE. 14.

8. Deery CB, Hanrahan CF, Selibas K, Bassett J, Sanne I, Van Rie A. A home tracing program for contacts of people with tuberculosis or HIV and patients lost to care. International Journal of Tuberculosis and Lung Disease. 18(5):534-40+i. PubMed PMID: 372973633.

9. Igbokwe CC, Abugu LI, Aji JO. Assessment of tuberculosis intensified case finding and isoniazid preventive therapy for people living with hiv in enugu state, nigeria. Afr J Biomed Res. 23(3):367-73.

10. Sah SK, Sahu SK, Lamichhane B, Bhatta GK, Bh, ari KB, et al. Dotting the Three I's for collaborative TB-HIV activities: Evaluation of a pilot programme in Kathmandu, Nepal. Public Health Action. 6(3):169-75. PubMed PMID: 612815398.

11. Tiam A, Machekano R, Gounder CR, Maama-Maime LBM, Ntene-Sealiete K, Sahu M, et al. Preventing tuberculosis among HIV-infected pregnant women in lesotho: The case for rolling out active case finding and isoniazid preventive therapy. Journal of Acquired Immune Deficiency Syndromes. 67(1):e5-e11. PubMed PMID: 373763298.

12. Costenaro P, Massavon W, Lundin R, Nabachwa SM, Fregonese F, Morelli E, et al. Implementation and Operational Research: Implementation of the WHO 2011 Recommendations for Isoniazid Preventive Therapy (IPT) in Children Living With HIV/AIDS: A Ugandan Experience. Journal of Acquired Immune Deficiency Syndromes: JAIDS. 71(1):e1-8. PubMed PMID: 26761275.

13. Peters JA, Heunis C, Kigozi G, Osoba T, Van Der Walt M. Integration of TB-HIV services at an anc facility in frances baard district, northern cape, south africa. Public Health Action. 2015;5(1):30-5. PubMed PMID: 603240996.

14. Kufa T, Fielding KL, Hippner P, Kielmann K, Vassall A, Churchyard GJ, et al. An intervention to optimise the delivery of integrated tuberculosis and HIV services at primary care clinics: results of the MERGE cluster randomised trial. Contemporary Clinical Trials. 72:43-52. PubMed PMID: 2000984591.

15. Carmone A, Rodriguez CA, Frank TD, Kiromat M, Bongi PW, Kuno RG, et al. Increasing isoniazid preventive therapy uptake in an HIV program in rural Papua New Guinea. Public Health Action. 7(3):193-8. PubMed PMID: 618478490.

16. Hunter OF, Kyesi F, Ahluwalia AK, Daffe ZN, Munseri P, von Reyn CF, et al. Successful implementation of isoniazid preventive therapy at a pediatric HIV clinic in Tanzania. BMC Infect Dis. 2020;20(1):738.

17. Adams LV, Mahlalela N, Talbot EA, Pasipamire M, Ginindza S, Calnan M, et al. High completion rates of isoniazid preventive therapy among persons living with HIV in Swaziland. International Journal of Tuberculosis & Lung Disease. 21(10):1127-32. PubMed PMID: 28911356.

18. Shayo GA, Moshiro C, Aboud S, Bakari M, Mugusi FM. Acceptability and adherence to Isoniazid preventive therapy in HIV-infected patients clinically screened for latent tuberculosis in Dar es Salaam, Tanzania. BMC Infectious Diseases. 15(368). PubMed PMID: 605749328.

19. Adepoju A, Ogbudebe C, Adejumo O, Okolie J, Inegbeboh J. Implementation of isoniazid preventive therapy among people living with HIV in Northwestern Nigeria: Completion rate and predictive factors. J Glob Infect Dis. 12(2):105-11.

20. Karanja M, Kingwara L, Owiti P, Kirui E, Ngari F, Kiplimo R, et al. Outcomes of isoniazid preventive therapy among people living with HIV in Kenya: A retrospective study of routine health care data. PLoS ONE. 2020;15(12):e0234588.
