# Supplementary material for: The latent tuberculosis cascade-of-care among people living with HIV: A systematic review and meta-analysis
Source: PLoS Med. 2021 Sep 7;18(9):e1003703. doi: 10.1371/journal.pmed.1003703 (PMC8439450; doi:10.1371/journal.pmed.1003703)
Supplement: S7 Table — (DOCX) [file pmed.1003703.s009.docx]

# S7 Table. Number of participants in each step of the cascade-of-care among studies that used LTBI tests.

| Study [ref] | Used LTBI test | Total identified (eligible for testing & treatment) | Initial LTBI test | Completed LTBI test | Need medical evaluation  (LTBI Test Positive) | Medical Evaluation | Recommended LTBI treatment | Started LTBI treatment | Completed LTBI treatment |
| --- | --- | --- | --- | --- | --- | --- | --- | --- | --- |
| Reported all steps of the cascade of care (all 6 proportions can be calculated) | | | | | | | | | |
| Cowger, Thai (1) | Yes | 659 | 359 | 359 | 81 | 81 | 81 | 74 | 36 |
| Mueller, Mpala (2) | Yes | 1021 | 741 | 701 | 331 | 331 | 264 | 263 | 253 |
| Kall, Coyne (3) | Yes | 600 | 520 | 502 | 50 | 50 | 46 | 40 | 38 |
| Khawcharoenporn, Phetsuksiri (4) | Yes | 150 | 150 | 150 | 36 | 36 | 20 | 16 | 15 |
| Aisu, Raviglione (5) | Yes | 1524 | 1344 | 1094 | 579 | 579 | 520 | 520 | 322 |
| Diaz, Diez (6) | Yes | 1242 | 853 | 846 | 181 | 181 | 142 | 122 | 70 |
| Marks, Taylor (7) | Yes | 109 | 80 | 73 | 11 | 11 | 11 | 11 | 6 |
| Capocci, Sewell (8) | Yes | 892 | 219 | 217 | 16 | 16 | 14 | 9 | 7 |
| Reported all steps up to starting LTBI treatment (proportions 1 through 5 calculated) | | | | | | | | | |
| Huerga, Mueller (9) | Yes | 897 | 576 | 499 | 366 | 366 | 204 | 196 |  |
| Doyle, Bissessor (10) | Yes | 1378 | 917 | 913 | 29 | 21 | 21 | 19 |  |
| Lee, Lee (11) | Yes | 474 | 464 | 464 | 93 | 93 | 93 | 40 |  |
| Rose, Kitai (12) | Yes | 81 | 81 | 81 | 15 | 15 | 15 | 7 |  |
| Lee, Lin (13) | Yes | 774 | 772 | 741 | 90 | 90 | 90 | 0 |  |
| Pullar, Steinum (14) | Yes | 304 | 304 | 298 | 77 | 77 | 64 | 39 |  |
| Reported all steps up to starting LTBI treatment (proportions 1 through 4 calculated) | | | | | | | | | |
| Aichelburg, Reiberger (15) | Yes | 1525 | 1506 | 1500 | 142 | 142 | 142 |  |  |
| Graves, Augusto (16) | Yes | 78 | 78 | 78 | 28 | 28 | 28 |  |  |
| Sandhu, Taylor (17) | Yes | 206 | 128 | 128 | 3 | 3 | 3 |  |  |
| Baker, Peterson (18) | Yes | 129 | 89 | 68 | 9 | 9 | 9 |  | 6 |
| Reported all steps up to medical evaluation (proportions 1 to 3 calculated) | | | | | | | | | |
| Bourgarit, Baron (19) | Yes | 506 | 415 | 415 | 66 | 66 |  | 13 |  |
| Xin, Li (20) | Yes | 184 | 182 | 182 | 16 | 16 |  |  |  |
| Reported all steps up to medical evaluation and later steps of the cascade (proportions 1 to 3, 5 and/or 6 calculated) | | | | | | | | | |
| Pascopella, Franks (21) | Yes | 500 | 393 | 377 | 20 | 19 |  | 14 | 3 |
| Leung, Chan (22) | Yes | 110 | 110 | 110 | 17 | 16 |  | 16 | 14 |
| Froberg, Jansson (23) | Yes | 69 | 39 | 39 | 6 | 6 |  | 0 | 0 |
| Santos, Garcia (24) | Yes | 872 | 759 | 690 | 66 |  | 53 | 39 | 26 |
| Reported only initial steps up to completed LTBI test (proportions 1 and 2 calculated) | | | | | | | | | |
| Al-Darraji, Kamarulzaman (25) | Yes | 189 | 140 | 138 | 117 |  |  |  |  |
| Reaves, Shah (26) | Yes | 2772 | 1907 | 1671 | 131 |  |  |  |  |
| Hirsch-Moverman, Cronin (27) | Yes | 71 | 53 | 35 | 9 |  |  |  |  |
| Fox-Lewis, Brima (28) | Yes | 165 | 20 | 20 |  |  |  |  |  |
| Sester, Van Leth (29) | Yes | 768 | 768 | 658 | 239 |  |  | 70 |  |
| Sun, Hsueh (30) | Yes | 617 | 608 | 598 | 64 |  |  | 0 |  |
| Zhang, Liu (31) | Yes | 50 | 50 | 50 | 21 |  |  | 0 |  |
| Reported all initial steps up to completed LTBI test and later steps of the cascade (proportions 1 and 2, and 5 or 6 calculated) | | | | | | | | | |
| Yang, Chan (32) | Yes | 1001 | 958 | 909 | 227 |  |  | 81 | 74 |
| Shin, Chang (33) | Yes | 15155 | 15125 | 15029 | 802 |  |  | 581 | 457 |
| Cheng, Hirji (34) | Yes | 129 | 105 | 64 | 39 |  |  | 9 | 1 |
| Missed initial steps of the cascade but reported middle and later steps of the cascade (proportions 2 though 6 calculated) | | | | | | | | | |
| Picone, Freitas (35) | Yes |  | 3260 | 2878 | 246 | 238 | 210 | 210 | 196 |
| Kussen, Dalla-Costa (36) | Yes |  | 154 | 140 | 34 | 13 | 13 | 13 | 13 |
| Monteiro, Guariente (37) | Yes |  | 220 | 188 | 9 | 9 | 6 | 3 | 1 |
| Missed initial steps of the cascade but reported middle and later steps of the cascade (proportions 3 though 6 calculated) | | | | | | | | | |
| Goletti, Navarra (38) | Yes | 774 |  | 507 | 33 | 32 | 19 | 16 | 8 |
| Missed initial steps of the cascade but reported middle steps of the cascade (proportions 3 and 4 calculated) | | | | | | | | | |
| Stein, Zalwango (39) | Yes | 199 |  | 197 | 142 | 142 | 142 |  |  |
| Meijerink, Wisaksana (40) | Yes |  |  | 524 | 151 | 151 | 151 |  |  |
| Missed initial steps of the cascade but reported middle steps of the cascade (proportion 3 calculated) | | | | | | | | | |
| Adams, Howe (41) | Yes | 372 |  | 118 | 1 | 1 |  |  |  |
| Brassard, Bruneau (42) | Yes |  |  | 60 | 3 | 1 |  |  |  |
| Missed initial steps of the cascade but reported later steps of the cascade (proportions 5 & 6 calculated) | | | | | | | | | |
| Golub, Cohn (43) | Yes | 17143 |  | 7361 | 2135 |  | 1954 | 1601 | 1330 |
| Lobato, Leary (44) | Yes | 4435 |  | 4355 | 1991 |  | 1238 | 1180 | 483 |
| Missed initial steps of the cascade but reported later steps of the cascade (proportion 5 calculated) | | | | | | | | | |
| Aquino, Moura (45) | Yes | 2341 |  | 1138 | 272 |  | 232 | 162 |  |
| Missed initial steps of the cascade but reported later steps of the cascade (proportion 6 calculated) | | | | | | | | | |
| Martinez-Pino, Sambeat (46) | Yes | 7923 |  | 2540 | 428 |  |  | 270 | 144 |
| Elzi, Schlegel (47) | Yes | 6160 |  | 4168 | 390 |  |  | 193 | 144 |
| Souza, Hökerberg (48) | Yes |  |  |  |  |  |  | 138 | 121 |
| No proportions can be calculated (only overall ratios) | | | | | | | | | |
| Wong, Leung (49) | Yes | 2079 |  | 1740 | 549 |  |  | 464 |  |

References

1. Cowger TL, Thai LH, Duong BD, Danyuttapolchai J, Kittimunkong S, Nhung NV, et al. Programmatic evaluation of an algorithm for intensified tuberculosis case finding and isoniazid preventive therapy for people living with HIV in Thailand and Vietnam. Journal of Acquired Immune Deficiency Syndromes. 2017;76(5):512-21. PubMed PMID: 621681989.

2. Mueller Y, Mpala Q, Kerschberger B, Rusch B, McHunu G, Mazibuko S, et al. Adherence, tolerability, and outcome after 36 months of isoniazid-preventive therapy in 2 rural clinics of Swaziland. Medicine (United States). 96. PubMed PMID: 618161560.

3. Kall MM, Coyne KM, Garrett NJ, Boyd AE, Ashcroft AT, Reeves I, et al. Latent and subclinical tuberculosis in HIV infected patients: a cross-sectional study. BMC Infect Dis. 12:107. PubMed PMID: 22558946.

4. Khawcharoenporn T, Phetsuksiri B, Rudeeaneksin J, Srisungngam S, Apisarnthanarak A. QuantiFERON-TB Gold In-Tube Test for Tuberculosis Prevention in HIV-Infected Patients. Japanese Journal of Infectious Diseases. 2017;70:502-6. PubMed PMID: r.

5. Aisu T, Raviglione MC, van Praag E, Eriki P, Narain JP, Barugahare L, et al. Preventive chemotherapy for HIV-associated tuberculosis in Uganda: an operational assessment at a voluntary counselling and testing centre. Aids. 9(3):267-73. PubMed PMID: 7755915.

6. Diaz A, Diez M, Bleda MJ, Aldamiz M, Camafort M, Camino X, et al. Eligibility for and outcome of treatment of latent tuberculosis infection in a cohort of HIV-infected people in Spain. BMC Infect Dis. 10:267. PubMed PMID: 20840743.

7. Marks SM, Taylor Z, Qualls NL, Shrestha-Kuwahara RJ, Wilce MA, Nguyen CH. Outcomes of contact investigations of infectious tuberculosis patients. Am J Respir Crit Care Med. 162(6):2033-8. PubMed PMID: 11112109.

8. Capocci SJ, Sewell J, Smith C, Cropley I, Bhagani S, Solamalai A, et al. Cost effectiveness of testing HIV infected individuals for TB in a low TB/HIV setting. J Infect Dis. 2020;81(2):289-96.

9. Huerga H, Mueller Y, Ferlazzo G, Mpala Q, Bevilacqua P, Vasquez B, et al. Implementation and Operational Research: Feasibility of Using Tuberculin Skin Test Screening for Initiation of 36-Month Isoniazid Preventive Therapy in HIV-Infected Patients in Resource-Constrained Settings. J Acquir Immune Defic Syndr. 2016;71(4):e89-95. Epub 2016/02/26. doi: 10.1097/qai.0000000000000895. PubMed PMID: 26910386.

10. Doyle JS, Bissessor M, Denholm JT, Ryan N, Fairley CK, Leslie DE. Latent tuberculosis screening using interferon-gamma release assays in an australian HIV-infected cohort: Is routine testing worthwhile? Journal of Acquired Immune Deficiency Syndromes. 66(1):48-54. PubMed PMID: 52976529.

11. Lee S, Lee JE, Kang JS, Lee SO, Lee SH. Long-term performance of the IGRA to predict and prevent active tuberculosis development in HIV-infected patients. International Journal of Tuberculosis and Lung Disease. 23(4):422-7. PubMed PMID: 2001979764.

12. Rose W, Kitai I, Kakkar F, Read SE, Behr MA, Bitnun A. Quantiferon Gold-in-tube assay for TB screening in HIV infected children: Influence of quantitative values. BMC Infectious Diseases. 14(516). PubMed PMID: 600063820.

13. Lee SS, Lin HH, Tsai HC, Su IJ, Yang CH, Sun HY, et al. A Clinical Algorithm to Identify HIV Patients at High Risk for Incident Active Tuberculosis: A Prospective 5-Year Cohort Study. PLoS ONE. 2015;10(8):e0135801. PubMed PMID: 26280669.

14. Pullar ND, Steinum H, Bruun JN, Dyrhol-Riise AM. HIV patients with latent tuberculosis living in a low-endemic country do not develop active disease during a 2 year follow-up; a Norwegian prospective multicenter study. BMC Infect Dis. 14:667. PubMed PMID: 25515915.

15. Aichelburg MC, Reiberger T, Breitenecker F, orfer M, Makristathis A, Rieger A. Reversion and conversion of interferon-gamma release assay results in HIV-1-infected individuals. J Infect Dis. 209(5):729-33. PubMed PMID: 23911707.

16. Graves SK, Augusto O, Viegas SO, Lederer P, David C, Lee K, et al. Tuberculosis infection risk, preventive therapy care cascade and incidence of tuberculosis disease in healthcare workers at Maputo Central Hospital. BMC Infectious Diseases. 19(1):346. PubMed PMID: 31023260.

17. Sandhu P, Taylor C, Miller RF, Post FA. Implementation of routine interferon-gamma release assay testing in a South London HIV cohort. Int J STD AIDS. 31(3):264-7.

18. Baker BJ, Peterson B, Mohanlall J, Singh S, Hicks C, Jacobs R, et al. Scale-up of collaborative TB/HIV activities in Guyana. Rev Panam Salud Publica. 2017;41:e6-e.

19. Bourgarit A, Baron G, Breton G, Tattevin P, Katlama C, Allavena C, et al. Latent tuberculosis infection screening and 2-year outcome in antiretroviral-naive HIV-infected patients in a low-prevalence country. Annals of the American Thoracic Society. 12(8):1138-45. PubMed PMID: 606143998.

20. Xin HN, Li XW, Zhang L, Li Z, Zhang HR, Yang Y, et al. Tuberculosis infection testing in HIV-positive men who have sex with men from Xi'an China. Epidemiology and Infection. 145(3):498-502. PubMed PMID: 613340710.

21. Pascopella L, Franks J, Marks SM, Salcedo K, Schmitz K, Colson PW, et al. Opportunities for tuberculosis diagnosis and prevention among persons living with HIV: A cross-sectional study of policies and practices at four large Ryan White program-funded HIV clinics. PLoS ONE. 9. PubMed PMID: 373528878.

22. Leung CC, Chan K, Yam WC, Lee MP, Chan CK, Wong KH, et al. Poor agreement between diagnostic tests for latent tuberculosis infection among HIV-infected persons in Hong Kong. Respirology. 2016. PubMed PMID: 610175911.

23. Froberg G, Jansson L, Nyberg K, Obasi B, Westling K, Berggren I, et al. Screening and treatment of tuberculosis among pregnant women in Stockholm, Sweden, 2016-2017. Eur Respir J. 2020;55(3):03.

24. Santos DTd, Garcia MC, Costa AANFd, Pieri FM, Meier DAP, Albanese SPR, et al. Infecção latente por tuberculose entre pessoas com HIV/AIDS, fatores associados e progressão para doença ativa em município no Sul do Brasil. Cad Saude Publica. 2017;33(8):e00050916-e.

25. Al-Darraji HA, Kamarulzaman A, Altice FL. Latent tuberculosis infection in a Malaysian prison: implications for a comprehensive integrated control program in prisons. BMC public health. 2014;14:22. PubMed PMID: 605670147.

26. Reaves EJ, Shah NS, France AM, Morris SB, Kammerer S, Skarbinski J, et al. Latent tuberculous infection testingamongHIV-infected persons in clinical care, United States, 2010-2012. International Journal of Tuberculosis and Lung Disease. 21(10):1118-26. PubMed PMID: 618346045.

27. Hirsch-Moverman Y, Cronin WA, Chen B, Moran JA, Munk E, Reichler MR. HIV counseling and testing in tuberculosis contact investigations in the United States and Canada. International Journal of Tuberculosis and Lung Disease. 19(8):943-53. PubMed PMID: 605446742.

28. Fox-Lewis A, Brima N, Muniina P, Grant AD, Edwards SG, Miller RF, et al. Tuberculosis screening in patients with HIV: An audit against UK national guidelines to assess current practice and the effectiveness of an electronic tuberculosis-screening prompt. International Journal of STD and AIDS. 27(10):901-5. PubMed PMID: 611908996.

29. Sester M, Van Leth F, Bruchfeld J, Bumbacea D, Cirillo DM, Dilektasli AG, et al. Risk assessment of tuberculosis in immunocompromised patients: A TBNET study. American Journal of Respiratory and Critical Care Medicine. 190(10):1168-76. PubMed PMID: 604384400.

30. Sun HY, Hsueh PR, Liu WC, Su YC, Chang SY, Hung CC, et al. Risk of Active Tuberculosis in HIV-Infected Patients in Taiwan with Free Access to HIV Care and a Positive T-Spot.TB Test. PLoS ONE. 2015;10(5):e0125260. PubMed PMID: 25938227.

31. Zhang LF, Liu XQ, Zuo LY, Li TS, Deng GH, Wang AX. Longitudinal observation of an interferon gamma-released assay (T-SPOT.TB) for Mycobacterium tuberculosis infection in AIDS patients on highly active antiretroviral therapy. Chin Med J (Engl). 123(9):1117-21. PubMed PMID: 20529548.

32. Yang CH, Chan PC, Liao ST, Cheng SH, Wong WW, Huang LM, et al. Strategy to better select HIV-infected individuals for latent TB treatment in BCG-vaccinated population. PLoS ONE. 2013;8(8):e73069. PubMed PMID: 24015285.

33. Shin SS, Chang AH, Ghosh JK, Dubé MP, Bolan R, Yang OO, et al. Isoniazid therapy for Mycobacterium tuberculosis infection in HIV clinics, Los Angeles, California. Int J Tuberc Lung Dis. 2016;20(7):961-6. Epub 2016/06/12. doi: 10.5588/ijtld.15.0988. PubMed PMID: 27287651; PubMed Central PMCID: PMCPMC4905690.

34. Cheng MP, Hirji A, Roth DZ, Cook VJ, Lima VD, Montaner JS, et al. Tuberculosis in HIV-infected persons in British Columbia during the HAART era. Canadian journal of public health = Revue canadienne de sante publique. 105(4):e258-e62. PubMed PMID: 603675458.

35. Picone CM, Freitas AC, Gutierrez EB, Avelino-Silva VI. Access and adherence to isoniazid preventive therapy and occurrence of active TB in a cohort of people living with HIV: a retrospective cohort study in Sao Paulo, Brazil. Rev Inst Med Trop Sao Paulo. 2020;62:e8.

36. Kussen GMB, Dalla-Costa LM, Rossoni A, Raboni SM. Interferon-gamma release assay versus tuberculin skin test for latent tuberculosis infection among HIV patients in Brazil. Braz J Infect Dis. 2016;20(1):69-75.

37. Monteiro ATA, Guariente MHDdM, Costa AANFd, Santos DTd, Alves E, Dessunti EM. Prova tuberculínica: o controle da tuberculose em pacientes infectados pelo vírus da imunodeficiência humana (HIV). Semin Ciênc Biol Saude. 2015;36(1):179-88.

38. Goletti D, Navarra A, Petruccioli E, Cimaglia C, Compagno M, Cuzzi G, et al. Latent tuberculosis infection screening in persons newly-diagnosed with HIV infection in Italy: a multicentre study promoted by the Italian Society of Infectious and Tropical Diseases. International journal of infectious diseases : IJID : official publication of the International Society for Infectious Diseases. 2019;27. PubMed PMID: 630481985.

39. Stein CM, Zalwango S, Malone LL, Thiel B, Mupere E, Nsereko M, et al. Resistance and Susceptibility to Mycobacterium tuberculosis Infection and Disease in Tuberculosis Households in Kampala, Uganda. American Journal of Epidemiology. 187(7):1477-89. PubMed PMID: 623483838.

40. Meijerink H, Wisaksana R, Lestari M, Meilana I, Chaidir L, Van Der Ven AJAM, et al. Active and latent tuberculosis among HIV-positive injecting drug users in Indonesia. Journal of the International AIDS Society. 18. PubMed PMID: 602273136.

41. Adams JW, Howe CJ, Andrews AC, Allen SL, Vinnard C. Tuberculosis screening among HIV-infected patients: tuberculin skin test vs. interferon-gamma release assay. AIDS Care - Psychological and Socio-Medical Aspects of AIDS/HIV. 29(12):1504-9. PubMed PMID: 616101560.

42. Brassard P, Bruneau J, Schwartzman K, Senecal M, Menzies D. Yield of tuberculin screening among injection drug users. Int J Tuberc Lung Dis. 8(8):988-93. PubMed PMID: 15305482.

43. Golub JE, Cohn S, Saraceni V, Cavalcante SC, Pacheco AG, Moulton LH, et al. Long-term protection from isoniazid preventive therapy for tuberculosis in HIV-infected patients in a medium-burden tuberculosis setting: the TB/HIV in Rio (THRio) study. Clin Infect Dis. 60(4):639-45. PubMed PMID: 25365974.

44. Lobato MN, Leary LS, Simone PM. Treatment for latent TB in correctional facilities: a challenge for TB elimination. Am J Prev Med. 24(3):249-53. PubMed PMID: 12657343.

45. Aquino DSd, Moura LCRV, Maruza M, Silva APd, Ximenes RAdA, Lacerda HR, et al. Factors associated with treatment for latent tuberculosis in persons living with HIV/AIDS. Cad Saude Publica. 2015;31(12):2505-13.

46. Martinez-Pino I, Sambeat MA, Lacalle-Remigio JR, Domingo P. Incidence of tuberculosis in HIV-infected patients in Spain: the impact of treatment for LTBI. Int J Tuberc Lung Dis. 17(12):1545-51. PubMed PMID: 24200266.

47. Elzi L, Schlegel M, Weber R, Hirschel B, Cavassini M, Schmid P, et al. Reducing tuberculosis incidence by tuberculin skin testing, preventive treatment, and antiretroviral therapy in an area of low tuberculosis transmission. Clin Infect Dis. 44(1):94-102. PubMed PMID: 17143823.

48. Souza CTVd, Hökerberg YHM, Pacheco S, Bedoya rJ, Rolla VC, Passos SRL. Effectiveness and safety of isoniazid chemoprophylaxis for HIV-1 infected patients from Rio de Janeiro. Mem Inst Oswaldo Cruz. 2009;104(3):462-7.

49. Wong NS, Leung CC, Chan KCW, Chan WK, Lin AWC, Lee SS. A longitudinal study on latent TB infection screening and its association with TB incidence in HIV patients. Sci Rep. 9. PubMed PMID: WOS:000475292700015.
