# Supplementary material for: The latent tuberculosis cascade-of-care among people living with HIV: A systematic review and meta-analysis
Source: PLoS Med. 2021 Sep 7;18(9):e1003703. doi: 10.1371/journal.pmed.1003703 (PMC8439450; doi:10.1371/journal.pmed.1003703)
Supplement: S8 Table — Pooled estimate for each step of the cascade-of-care, using random effect model. (DOCX) [file pmed.1003703.s010.docx]

|  | All cohorts | | | | Cohorts that reported all cascade-of-care steps | |
| --- | --- | --- | --- | --- | --- | --- |
| Steps | **Cohorts** | **n/N** | **Pooled estimate (95 CI%)** | **Pooled estimate (95 CI%) of cumulative percentage retained in the cascade** | **Cohorts** | **Calculated cumulative percentage retained in the cascade (95 CI%)^1^** |
| *Did not use LTBI tests (n=21 cohorts)^2^* | | | | | | |
| Proportion 1: Had a medical evaluation /Identified | 15 | 10806/13552 | 92.6% (95% CI: 75.8% to 98.0%) | 92.6% (95% CI: 75.8% to 98.0%) | 6 | 97.3% (95% CI: 46.4% to 99.9%) |
| Proportion 2: Recommended TPT /med evaluation | 10 | 5464/7040 | 80.9% (95% CI: 68.3% to 89.2%) | 74.9% (95% CI: 51.8% to 87.4%) | 6 | 76.3% (95% CI: 27.5% to 89.9%) |
| Proportion 3: Started TPT /Recommended TPT | 10 | 3259/4922 | 88.5% (95% CI: 34.1% to 99.1%) | 66.3% (95% CI: 17.7% to 86.6%) | 6 | 59.0% (95% CI: 4.7% to 88.4%) |
| Proportion 4: Completed TPT treatment/Started TPT | 13 | 8064/9937 | 81.5% (95% CI: 71.1% to 88.8%) | 54.0% (95% CI: 12.6% to 76.9%) | 6 | 42.6% (95% CI: 2.6% to 74.5%) |
| *Used LTBI tests (n=49 cohorts)* | | | | | | |
| Proportion 1: Initiated LTBI testing/Identified | 34 | 30813/35201 | 93.9% (95% CI: 93.8% to 94.0%) | 93.9% (95% CI: 93.8% to 94.0%) | 8 | 79..4% (95% CI: 53.4% to 92.9%) |
| Proportion 2: Completed LTBI testing/initiated LTBI test | 37 | 32899/34447 | 98.0% (95% CI: 96.0% to 99.0%) | 92.0% (95% CI: 90.1% to 93.1%) | 8 | 78.1% (95% CI: 50.0% to 92.5%) |
| *Prevalence of LTBI positive: Tests positive/completed test* | 47^3^ | 10131/55587 | 17.6% (95% CI: 13.3% to 23.0%) | - |  | - |
| Proportion 3: Medical evaluation completed /Needed medical evaluation | 31 | 2839/2881 | 99.9% (95% CI: 99%.0 to 100%) | 91.9 % (95% CI: 89.1% to 93.1%) | 8 | 77.5% (95% CI: 49.1% to 92.3%)^4^ |
| Proportion 4: Recommended TPT/Medical evaluation completed | 24 | 2308/2714 | 98.1% (95% CI: 91.8% to 99.6%) | 90.2% (95% CI: 81.8% to 92.7%) | 8 | 69.5% (95% CI: 37.3% to 88.1%) |
| Proportion 5: Started TPT /Recommended LTBI treatment | 23 | 4583/5313 | 89.7% (95% CI: 73.0% to 96.6%) | 80.9% (95% CI: 60.5% to 89.5%) | 8 | 66.9% (95% CI: 30.6% to 88.0%) |
| Proportion 6: Completed TPT /Started TPT treatment | 23 | 3762/5419 | 74.5% (95% CI: 62.4% to 83.8%) | 60.3% (95% CI:37.7% to 75.0%) | 8 | 53.1% (95% CI: 18.4% to 79.9%) |
| Notes: 1-This value is the product of the cumulative percentage from the preceding step, multiplied by the pooled estimate from this step. 2 All cohorts from low middle-income countries. 3.Among 49 cohorts, 47 reported the positivity rates of LTBI tests.4- Confidence intervals estimated using inverse method. Abbreviations: CI: Confidence interval, N: Number, TPT: Tuberculosis Preventive Therapy | | | | | | |

# S8 Table. Sensitivity analysis. Pooled estimate for each step of the cascade-of-care, **using random effect model^1^**
