# Supplementary material for: The latent tuberculosis cascade-of-care among people living with HIV: A systematic review and meta-analysis
Source: PLoS Med. 2021 Sep 7;18(9):e1003703. doi: 10.1371/journal.pmed.1003703 (PMC8439450; doi:10.1371/journal.pmed.1003703)
Supplement: S9 Table — Pooled estimate for each step of the cascade in cohorts that used LTBI tests, stratified by country income level1 random effect model. (DOCX) [file pmed.1003703.s011.docx]

# S9 Table. Sensitivity analysis Table. Pooled estimate for each step of the cascade in cohorts that used LTBI tests, stratified by country income level1**^-^ random effect model**

| Steps | Cohorts | n/N | Pooled estimate | Calculated cumulative percentage retained in the cascade (95 CI%)^2,^ |
| --- | --- | --- | --- | --- |
| *High income countries (N=25 cohorts)* | | | | |
| Proportion 1: Initiated LTBI testing/Identified | 19 | 23239/26288 | 89.7% (95% CI: 72.9% to 96.5%) | 89.7% (95% CI: 72.9% to 96.5%) |
| Proportion 2: Completed LTBI testing/initiated LTBI test | 19 | 22778/23239 | 98.8% (95% CI: 96.5% to 99.6%) | 88.6% (95% CI: 70.3% to 96.1%) |
| *Prevalence of LTBI test positive: Positive/completed test* | 24 | 4551/34506 | 11.6% (95% CI: 8.0% to 16.6%) | - |
| Proportion 3: Medical evaluation completed /Needed medical evaluation | 17 | 750/763 | 99.7% (95% CI: 96% to 100%) | 88.4% (95% CI: 67.5% to 96.1%) |
| Proportion 4: Recommended TPT/Medical evaluation completed | 11 | 570/641 | 97.6% (95% CI: 84.9% to 99.7%) | 86.2% (95% CI: 57.3% to 95.8%) |
| Proportion 5: Started TPT /Recommended LTBI treatment | 11 | 1486/1666 | 81.8% (95% CI: 67.6% to 90.6%) | 70.5% (95% CI: 38.8% to 86.8%) |
| Proportion 6: Completed TPT /Started TPT | 12 | 1375/2461 | 61.9% (95% CI: 45.4% to 76%) | 43.7% (95% CI: 17.6% to 66.0%) |
| *Low- and middle-income countries (N=23 cohorts)* | | | | |
| Proportion 1: Initiated LTBI testing/Identified | 14 | 6806/8145 | 95.4% (95% CI: 85.6% to 98.7%) | 95.4% (95% CI: 85.6% to 98.7%) |
| Proportion 2: Completed LTBI testing/initiated LTBI test | 17 | 9463/10440 | 97.0% (95% CI: 92.6% to 98.8%) | 92.5% (95% CI:79.3% to 97.5%) |
| *Prevalence of LTBI test positive: Positive/completed test* | 22 | 5341/20423 | 25.8% (95% CI: 18.1% to 35.3%) | - |
| Proportion 3: Medical evaluation completed /Needed medical evaluation | 14 | 2089/2118 | 100% (95% CI: 31.8% to 100%) | 92.5% (95% CI: 25.2% to 97.5%) |
| Proportion 4: Recommended TPT/Medical evaluation completed | 13 | 1738/2073 | 98.4% (95% CI: 86.3% to 99.8%) | 91.1% (95% CI: 21.8% to 97.3%) |
| Proportion 5: Started TPT /Recommended LTBI treatment | 12 | 3097/3647 | 93.9% (95% CI: 58.4% to 99.4%) | 85.5% (95% CI: 12.7% to 96.7%) |
| Proportion 6: Completed TPT /Started TPT | 11 | 2387/2958 | 84.7% (95% CI: 72.7% to 92.1%) | 72.4% (95% CI: 9.2% to 89.1%) |
| Notes:  1-One multicenter study, in different countries with different income classification, not included in these analyses since the cascade steps were not reported by center (*Sester* *M* al [1])  2-This value is the product of the cumulative percentage from the preceding step, multiplied by the pooled estimate from this step.  Abbreviations: CI: Confidence interval, LTBI: latent tuberculosis infection, N: Number, TPT : Tuberculosis Preventive Therapy | | | | |

Reference

1. Sester M, Van Leth F, Bruchfeld J, Bumbacea D, Cirillo DM, Dilektasli AG, et al. Risk assessment of tuberculosis in immunocompromised patients: A TBNET study. American Journal of Respiratory and Critical Care Medicine. 190(10):1168-76. PubMed PMID: 604384400.
