# Supplementary material for: The latent tuberculosis cascade-of-care among people living with HIV: A systematic review and meta-analysis
Source: PLoS Med. 2021 Sep 7;18(9):e1003703. doi: 10.1371/journal.pmed.1003703 (PMC8439450; doi:10.1371/journal.pmed.1003703)
Supplement: S10 Table — (DOCX) [file pmed.1003703.s012.docx]

# S10 Table-Summary results of the report U.S. President’s Emergency Plan for AIDS Relief, 2017–2019 (PEPFAR) Tuberculosis Preventive Treatment Scale-Up Among Antiretroviral Therapy Patients [1]

| Country | N of PLHIV started TPT | N of PLHIV completed TPT (% started TPT) |
| --- | --- | --- |
| Cameroon | 10,187 | 4,253 (41.7%) |
| Democratic Republic of the Congo | 53,859 | 34,752 (64.5%) |
| Eswatini | 32,001 | 24,954 (78.0%) |
| Ethiopia | 44,988 | 36,177 (80.4%) |
| Haiti | 26,510 | 14,798 (55.8%) |
| Kenya | 287,258 | 231,804 (80.7%) |
| Lesotho | 13,686 | 2,984 (21.8%) |
| Mozambique | 266,482 | 56,306 (21.1%) |
| Namibia | 22,029 | 19,681 (89.3%) |
| Nigeria | 259,438 | 202,174 (77.9%) |
| South Africa | 385,725 | 184,529 (47.8%) |
| Tanzania | 250,291 | 155,535 (62.1%) |
| Uganda | 44,958 | 31,121 (69.2%) |
| Vietnam | 18,126 | 14,015 (77.3%) |
| Zambia | 65,852 | 47,674 (72.4%) |
| Zimbabwe | 23,758 | 18,114 (76.2%) |
| Total | **1,805,148** | **1,078,871 (59.8%)** |
| Abbreviation: PLHIV: patient living with HIV, TPT: tuberculosis preventive therapy | | |

Reference

1. Melgar M, Nichols C, Cavanaugh JS, Kirking HL, Surie D, Date A, et al. Tuberculosis Preventive Treatment Scale-Up Among Antiretroviral Therapy Patients - 16 Countries Supported by the U.S. President's Emergency Plan for AIDS Relief, 2017-2019. MMWR Morb Mortal Wkly Rep. 2020;69(12):329-34.
