# Supplementary material for: The latent tuberculosis cascade-of-care among people living with HIV: A systematic review and meta-analysis
Source: PLoS Med. 2021 Sep 7;18(9):e1003703. doi: 10.1371/journal.pmed.1003703 (PMC8439450; doi:10.1371/journal.pmed.1003703)
Supplement: S11 Table — (DOCX) [file pmed.1003703.s013.docx]

# S11 Table. Meta regression of patients completing TPT over PLHIV identified.^1^

| Meta-analyses | Pooled estimates | - |
| --- | --- | --- |
| TPT completion/PLHIV identified | 26.5% (95% CI: 18.9% to 35.9%) |  |
| *Meta-regression* | **Odds ratio (95% CI)** | **p-value** |
| LTBI tests |  |  |
| Did not use LTBI test | Reference |  |
| Used LTBI tests | 1.5 (95% CI 0.6 to 3.7) | 0.3830 |
| Income |  |  |
| Low -middle income | Reference |  |
| High income | 0.8 (95% CI 0.4 to 2.2) | 0.7845 |
| Type of service |  |  |
| HIV care | Reference |  |
| Other clinics | 1.4 (0.5 to 3.5) | 0.4376 |
| 1-Among the studies that used LTBI tests, PLHIV identified were multiplied by the prevalence of LTBI test positive. | | |
