# Supplementary material for: The latent tuberculosis cascade-of-care among people living with HIV: A systematic review and meta-analysis
Source: PLoS Med. 2021 Sep 7;18(9):e1003703. doi: 10.1371/journal.pmed.1003703 (PMC8439450; doi:10.1371/journal.pmed.1003703)

# S1 Fig. Quality assessment of the studies included in the review

1. Summary of quality assessment


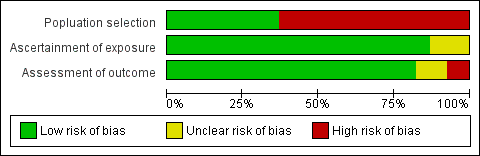


1. Summary for individual studies


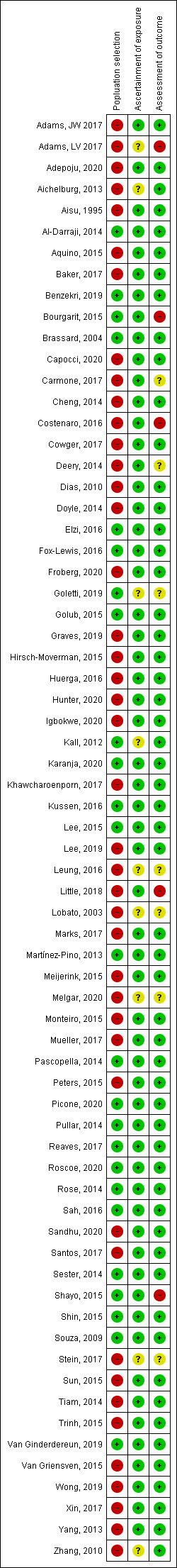

Supplement: S1 Fig — (DOCX) [file pmed.1003703.s014.docx]
