# Supplementary material for: The latent tuberculosis cascade-of-care among people living with HIV: A systematic review and meta-analysis
Source: PLoS Med. 2021 Sep 7;18(9):e1003703. doi: 10.1371/journal.pmed.1003703 (PMC8439450; doi:10.1371/journal.pmed.1003703)
Supplement: S2 Fig — LTBI, latent tuberculosis infection; TPT, tuberculosis preventive therapy. (DOCX) [file pmed.1003703.s015.docx]

# S2 Fig. Forest plots among studies that did not use LTBI tests

b)

c)

d)
