# Supplementary material for: The latent tuberculosis cascade-of-care among people living with HIV: A systematic review and meta-analysis
Source: PLoS Med. 2021 Sep 7;18(9):e1003703. doi: 10.1371/journal.pmed.1003703 (PMC8439450; doi:10.1371/journal.pmed.1003703)

# S4 Fig. Cumulative proportion for each step of the cascade in cohorts that used LTBI tests, stratified by type of LTBI test used (N=49 cohorts). Pooled using fixed effect model


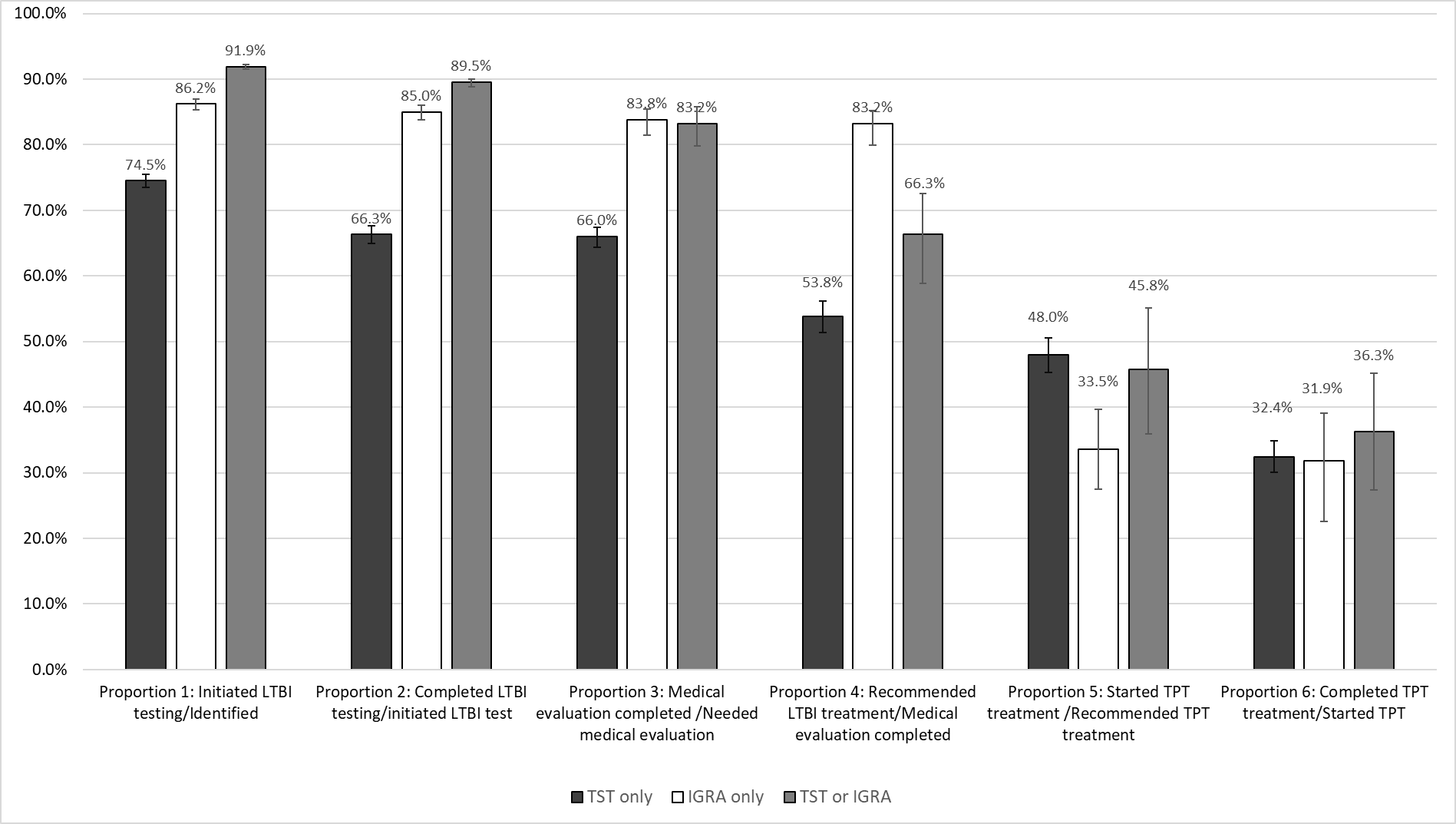

Supplement: S4 Fig — Pooled using fixed effect model. IGRA, interferon gamma release assay; LTBI, latent tuberculosis infection; PLHIV, people living with HIV; TPT, tuberculosis preventive therapy; TST, tuberculin skin test. (DOCX) [file pmed.1003703.s017.docx]
