# Supplementary material for: The latent tuberculosis cascade-of-care among people living with HIV: A systematic review and meta-analysis
Source: PLoS Med. 2021 Sep 7;18(9):e1003703. doi: 10.1371/journal.pmed.1003703 (PMC8439450; doi:10.1371/journal.pmed.1003703)

# S5 Fig. Cumulative proportion of each step of the cascade among cohorts that did not use LTBI test stratified by type of clinic where PLHIV were evaluated (N=21 cohorts). Pooled using fixed effect model


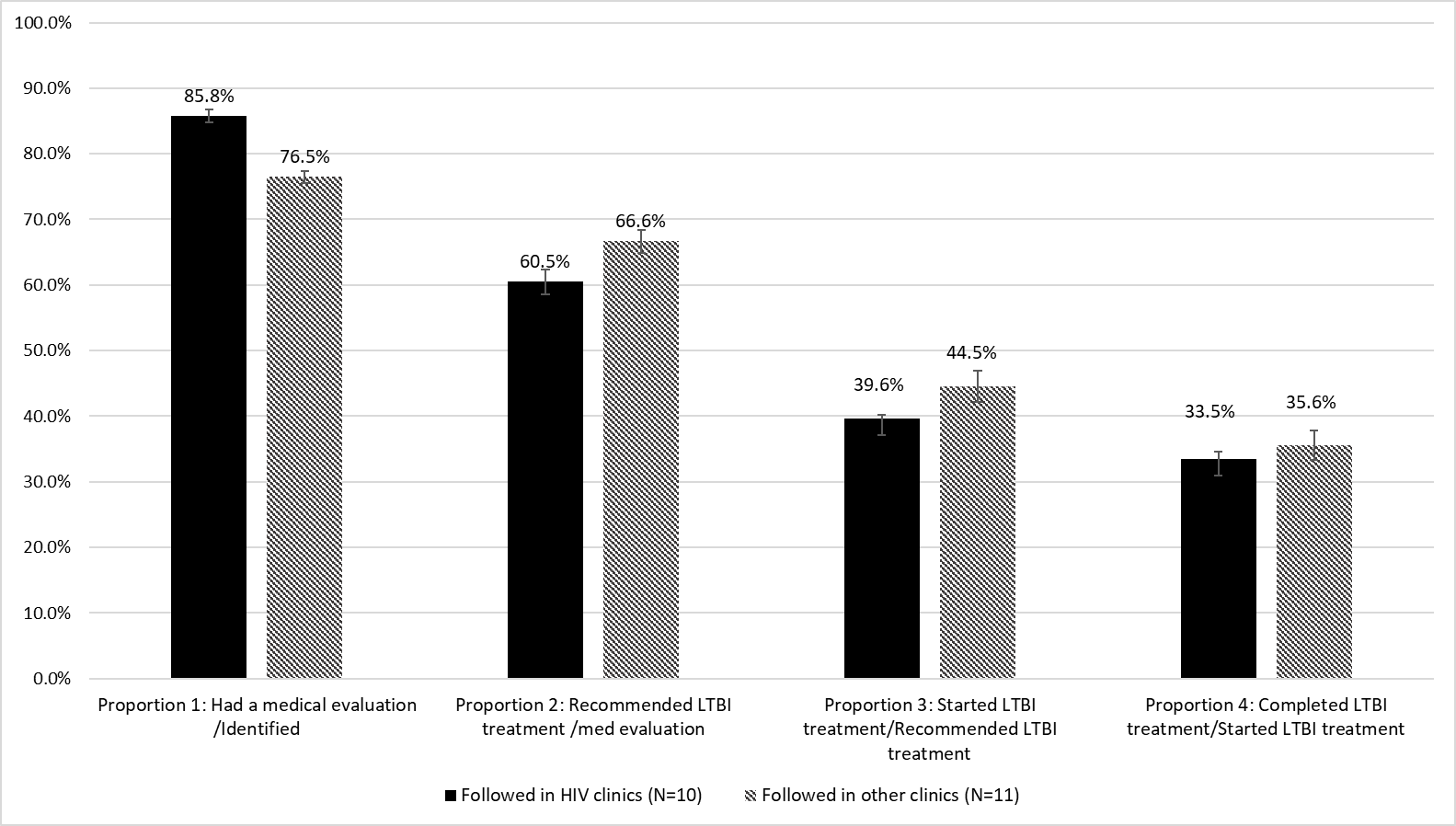

Supplement: S5 Fig — Pooled using fixed effect model. LTBI, latent tuberculosis infection; PLHIV, people living with HIV. (DOCX) [file pmed.1003703.s018.docx]
