# Supplementary material for: The latent tuberculosis cascade-of-care among people living with HIV: A systematic review and meta-analysis
Source: PLoS Med. 2021 Sep 7;18(9):e1003703. doi: 10.1371/journal.pmed.1003703 (PMC8439450; doi:10.1371/journal.pmed.1003703)

# S6 Fig. Cumulative proportion of each step of the cascade among cohorts that used LTBI test stratified by type of clinic where PLHIV were evaluated (N=49 cohorts) – Pooled using fixed effect model


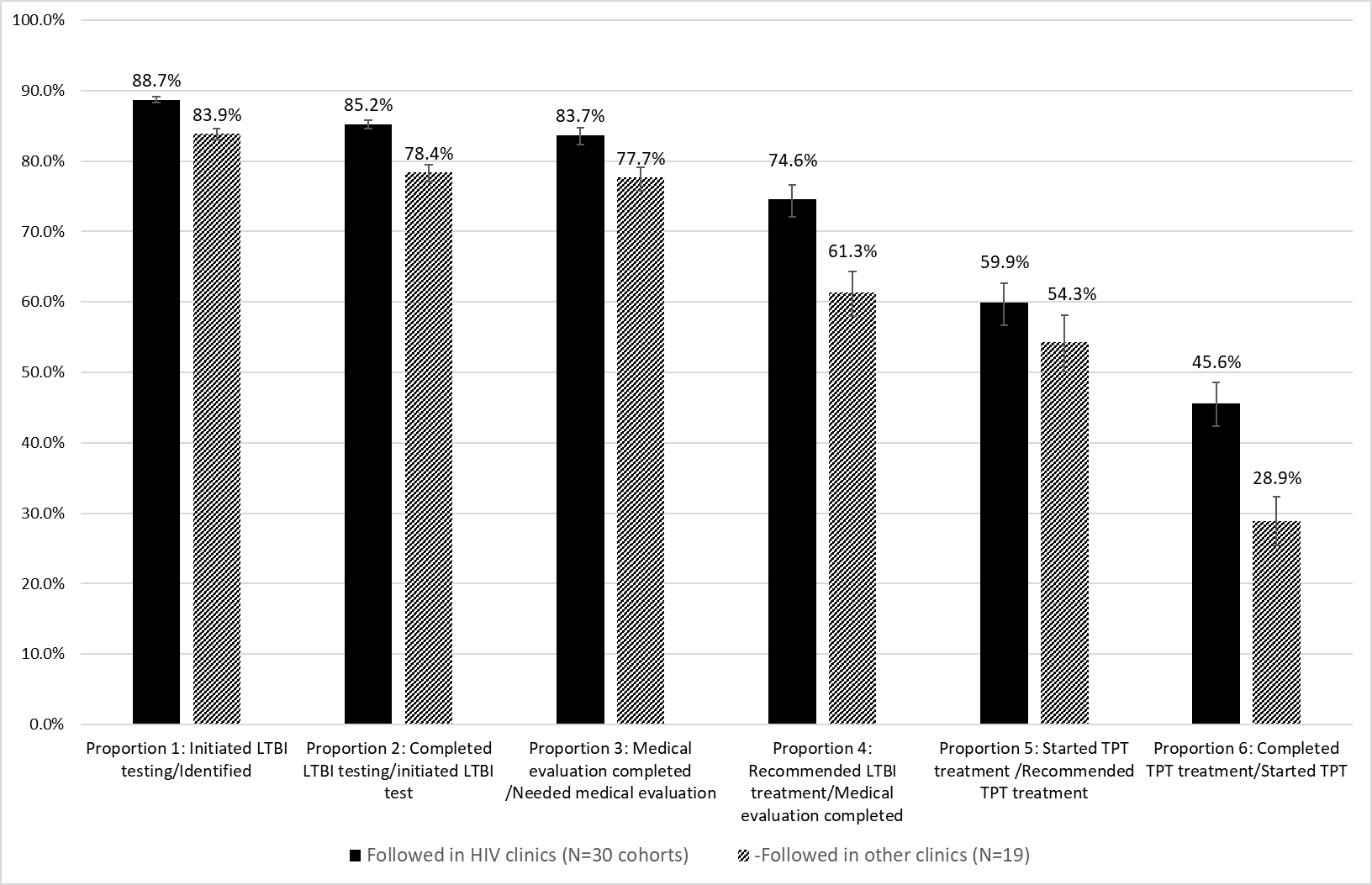

Supplement: S6 Fig — Pooled using fixed effect model. LTBI, latent tuberculosis infection; PLHIV, people living with HIV; TPT, tuberculosis preventive therapy. (DOCX) [file pmed.1003703.s019.docx]
