# Supplementary material for: The latent tuberculosis cascade-of-care among people living with HIV: A systematic review and meta-analysis
Source: PLoS Med. 2021 Sep 7;18(9):e1003703. doi: 10.1371/journal.pmed.1003703 (PMC8439450; doi:10.1371/journal.pmed.1003703)
Supplement: Attachment — Submitted filename: bastos.pdf [file pmed.1003703.s021.pdf]

This paper reports a systematic review with meta–analysis of studies of the tuberculosis cascade of care.

I was asked for a statistical report and I interpret that to include all aspects of the design and conduct of the study.

Points of detail

Page 2 The sentence starting 'Completion was similar' seems to have either an omission or an insertion.

Page 4 A brief scan of the reference list suggests all the article retrieved were published in English. Various empirical studies have indicated that the main bibliographic databases have varied and incomplete coverage of material not in English (Pilkington et al., 2005), and have limited coverage of material from low and middle–income country journals (Kieling et al., 2009; Syed Sheriff et al., 2008). It might be worth searching some more specialist databases like the WHO sponsored ones.

Page 4 That list of languages should cover the vast majority of studies but later could we have a report on whether any in other languages had to be omitted?

Page 5 Ideally one would measure agreement using a chance–corrected measure like Cohen's κ especially if the marginal frequencies were unbalanced. Having said that Table 1 suggests that most of the categories were not too unbalanced so it may not make much difference.

Page 6 As well as describing how the proportions were cascaded the method for propagating uncertainty to obtain the confidence intervals could usefully be included here.

Page 6 I think we could use some more detail about the choice of model here. While I agree that this can be done using a generalised linear mixed model the reader is going to have to investigate much further to find out that this is a model for the log odds. At least that is what I think `meta` does, I do not use it myself. The reader is also going to be surprised to find the authors choosing a fixed effect model for epidemiological data where considerable heterogeneity is almost guaranteed. It is possible to justify this (Rice et al., 2018) but I think we need to see the authors' reasoning here.

Pages 10 to 12 I realise there are many analyses summarised here but I still think we need more detail. Since the authors already have the

data stored it should be possible to generate forest plots which could go into the supplementary material so that we can see how variable the studies were and whether there are any outliers or influential studies.

Page 13 For the stratified analyses why not perform a formal test for whether the strata differ?

Figure 1b I think there are a couple of typos here: Proportion 3 and Proportion 4 texts look to have been truncated.

Summary

A lot of work went into this but I feel it is rather under–reported. I have indicated a couple of places where more detail would be beneficial.

Michael Dewey

References

C Kieling, H Herrman, V Patel, and J J Mari. Indexation of psychiatric journals from low– and middle–income countries: a survey and a case study. *World Psychiatry*, 8:40–44, 2009.

K Pilkington, A Boshnakova, M Clarke, and J Richardson. 'No language restrictions' in database searches: what does this really mean? *The Journal of Alternative and Complementary Medicine*, 11:205–207, 2005.

K Rice, J P T Higgins, and T Lumley. A re–evaluation of fixed effect(s) meta–analysis. *Journal of the Royal Statistical Society*, 181:205–227, 2018.

R J Syed Sheriff, C E Adams, P Tharyan, M Jayaram, L Duley, and the PRACTIHC Mental Health Group. Randomised trials relevant to mental health conducted in low and middle–income countries: a survey. *BMC Psychiatry*, 8(69), 2008.
