# Supplementary material for: The latent tuberculosis cascade-of-care among people living with HIV: A systematic review and meta-analysis
Source: PLoS Med. 2021 Sep 7;18(9):e1003703. doi: 10.1371/journal.pmed.1003703 (PMC8439450; doi:10.1371/journal.pmed.1003703)
Supplement: Attachment — Submitted filename: Cascade TPTinHIV ResponsetoReviewers.docx [file pmed.1003703.s022.docx]

Montreal, 31 March 2021

*REF: “The Latent Tuberculosis Cascade-of-Care Among People Living with HIV: A Systematic Review and Meta-Analysis" (PMEDICINE-D-20-06012R1)”*

Dear Dr. Richard Turner,

*Many thanks for your letter of February 17^th^, 2020, regarding the above titled manuscript.*

*We have revised our manuscript extensively, based on your instructions, and the reviewers’ comments, and have also prepared a point-by-point response – indicating where we have agreed, and occasionally, disagreed (in blue below). Note the line number refer to the CLEAN text. We also submitted the marj=ked up text - in case needed. And for the Figures - they ar submitted as separate TIF files, but also in one word file with all legends and titles - may be easier to read for you and reviewers.*

*We hope the revised manuscript is now acceptable for publication in PLOS Medicine, and look forward to hearing from you.*

*Yours very truly,*

*Dick Menzies and Mayara Bastos,*

*On behalf of all authors*

***Editorial requests***

1-In revising the manuscript for further consideration, your revisions should address the specific points made by each reviewer and the editors. Please also check the guidelines for revised papers at [http://journals.plos.org/plosmedicine/s/revising-your-manuscript](http://journals.plos.org/plosmedicine/s/revising-your-manuscript%20) for any that apply to your paper. In your rebuttal letter you should indicate your response to the reviewers' and editors' comments, the changes you have made in the manuscript, and include either an excerpt of the revised text or the location (eg: page and line number) where each change can be found. Please submit a clean version of the paper as the main article file; a version with changes marked should be uploaded as a marked up manuscript.

R: Please see below our responses to all comments and suggestions. We are submitting a clean and marked up copy of the manuscript.

2-In addition, we request that you upload any figures associated with your paper as individual TIF or EPS files with 300dpi resolution at resubmission; please read our figure guidelines for more information on our requirements: [http://journals.plos.org/plosmedicine/s/figures.](http://journals.plos.org/plosmedicine/s/figures.%20) While revising your submission, please upload your figure files to the PACE digital diagnostic tool, [https://pacev2.apexcovantage.com/.](https://pacev2.apexcovantage.com/.%20) PACE helps ensure that figures meet PLOS requirements. To use PACE, you must first register as a user. Then, login and navigate to the UPLOAD tab, where you will find detailed instructions on how to use the tool. If you encounter any issues or have any questions when using PACE, please email us at [PLOSMedicine@plos.org.](mailto:PLOSMedicine@plos.org.)

R: All submitted figures followed the above guidelines.

3-We hope to receive your revised manuscript by Mar 10 2021 11:59PM. Please email us ([plosmedicine@plos.org](mailto:plosmedicine@plos.org)) if you have any questions or concerns. ***Please note while forming your response, if your article is accepted, you may have the opportunity to make the peer review history publicly available. The record will include editor decision letters (with reviews) and your responses to reviewer comments. If eligible, we will contact you to opt in or out.***

R: As per previous email communications, we are submitting our revised manuscript by March 31^st^.

4-We ask every co-author listed on the manuscript to fill in a contributing author statement, making sure to declare all competing interests. If any of the co-authors have not filled in the statement, we will remind them to do so when the paper is revised. If all statements are not completed in a timely fashion this could hold up the re-review process. If new competing interests are declared later in the revision process, this may also hold up the submission. Should there be a problem getting one of your co-authors to fill in a statement we will be in contact.  YOU MUST NOT ADD OR REMOVE AUTHORS UNLESS YOU HAVE ALERTED THE EDITOR HANDLING THE MANUSCRIPT TO THE CHANGE AND THEY SPECIFICALLY HAVE AGREED TO IT. You can see our competing interests’ policy here: <http://journals.plos.org/plosmedicine/s/competing-interests>.

R: All authors have completed the “contributing author statement”.

5-To enhance the reproducibility of your results, we recommend that you deposit your laboratory protocols in protocols.io, where a protocol can be assigned its own identifier (DOI) such that it can be cited independently in the future. For instructions see <http://journals.plos.org/plosmedicine/s/submission-guidelines#loc-methods>.

R: Our protocol is submitted in PROSPERO (CRD42020190264).

6- Please ensure that the paper adheres to the PLOS Data Availability Policy (see <http://journals.plos.org/plosmedicine/s/data-availability>), which requires that all data underlying the study's findings be provided in a repository or as Supporting Information. For data residing with a third party, authors are required to provide instructions with contact information for obtaining the data. PLOS journals do not allow statements supported by "data not shown" or "unpublished results." For such statements, authors must provide supporting data or cite public sources that include it.

R: All the data used in our meta-analyses is available in the supplementary material

***Requests from the editors:***

7-Please resubmit your article as a "Research article" (which is standard for systematic reviews and meta-analyses at PLOS Medicine), and we will assign an academic editor to the paper upon resubmission.

R: Done.

8-Please update the search, e.g., to the start of 2021.

R: We have updated the search up to 17 Feb 2021, and we have included a new database (LILACS) as suggested by the reviewer 1. We included 17 new cohorts in the update review.

9-Please combine the "Methods" and "Findings" subsections of your abstract. The final sentence of the new combined subsection should begin "Study limitations include ..." or similar and should quote 2-3 of the study's main limitations.

R: Done.

10-Please quote some information on the cohorts in the abstract, e.g., the number or proportion in high-income countries and so on; and on the designs of the included studies. Are you able to quote aggregate demographic details for participants?

R: Done.

11-After the abstract, please add a new and accessible "author summary" section in non-identical prose. You may find it helpful to consult one or two recent research articles published in PLOS Medicine to get a sense of the preferred style.

R: Done.

12-Please revisit "was better" at line 222, explaining in a sentence, say, the respects in which coverage was "better" and perhaps quoting quantitative findings. We also suggest reiterating the patient group in this sentence.

R: We revised the paragraph, as suggested by the editorial group and the reviewer 2.

13-In your Discussion section, please reverse the order of the "strengths" and "limitations" paragraphs.

R: Thank you for your suggestion. We revised the discussion, and we revised the discussion, and re-organized the discussion into summary of findings, implications, limitations and strengths. As suggested by reviewer 2.

14- Throughout the text, please style reference call-outs as follows: " ... one cohort [17,66]." (i.e., no spaces within the square brackets). Please ensure that call-outs fall before punctuation, e.g., at line 223.

R: We revised all text to ensure that the citations meet the editorial guidelines.

15- In the reference list, please abbreviate journal names consistently (e.g., "Lancet" will suffice for reference 2). Please remove extraneous information on competing interests, e.g., from reference 9.

R: We revised the entire bibliography to ensure that the references meet the editorial guidelines.

16- Please remove the attached Collection proposal form.

R: Done.

 17-Please supply a completed PRISMA checklist with your revision, labelled "S1_PRISMA_Checklist" or similar and referred to in the Methods section of your main text. In the checklist, please refer to individual items by section (e.g., "Methods") and paragraph number rather than by page or line numbers, as the latter generally change in the event of publication.

R: We apologize for not submitting the PRISMA in the original submission. We are submitting this with the revised version as requested.

***Comments from the reviewers:***

**Reviewer #1:**

This paper reports a systematic review with meta-analysis of studies of the tuberculosis cascade of care. I was asked for a statistical report and I interpret that to include all aspects of the design and conduct of the study. Summary: A lot of work went into this, but I feel it is rather underreported. I have indicated a couple of places where more detail would be beneficial.

Points of detail

18- Page 2 The sentence starting `Completion was similar' seems to have either an omission or an insertion.

R: Thank you for pointing that out, we revised the sentence.

19- Page 4 A brief scan of the reference list suggests all the article retrieved were published in English. Various empirical studies have indicated that the main bibliographic databases have varied and incomplete coverage of material not in English (Pilkington et al., 2005), and have limited coverage of material from low and middle-income country journals (Kieling et al., 2009; Syed Sheri_ et al., 2008). It might be worth searching some more specialist databases like the WHO sponsored ones.

R: We have added one WHO sponsored database – LILACS, from the inception of this database up to Feb 17, 2021. We used a combination of terms in English, Spanish and Portuguese (Available in supplementary material). We found 707 titles and included 7 new full texts in our review, from this LILACS database.

20-Page 4 That list of languages should cover the vast majority of studies but later could we have a report on whether any in other languages had to be omitted?

R: No paper was excluded by language criteria. We added a sentence stating this in the results - Lines 216-217.

21-Page 5 Ideally one would measure agreement using a chance-corrected measure like Cohen's especially if the marginal frequencies were unbalanced. Having said that Table 1 suggests that most of the categories were not too unbalanced so it may not make much difference.

R: We felt that reporting the simple percentage agreement would be enough.

22-Page 6 As well as describing how the proportions were cascaded the method for propagating uncertainty to obtain the confidence intervals could usefully be included here.

R: We added an explanation in methods section (lines 181-182)

23- Page 6 I think we could use some more detail about the choice of model here. While I agree that this can be done using a generalised linear mixed model the reader is going to have to investigate much further to find out that this is a model for the log odds. At least that is what I think meta does, I do not use it myself. The reader is also going to be surprised to find the authors choosing a fixed effect model for epidemiological data where considerable heterogeneity is almost guaranteed. It is possible to justify this (Rice et al., 2018) but I think we need to see the authors' reasoning here.

R: We have added more details on the meta-analysis’s method (line 174-185). We also agree that the choice of presenting the fixed effect model can be questioned due to the heterogeneity of studies. However, when we displayed the cascade proportions graphically, the overlap of confidence intervals made it more difficult to visually present the results. So, we have presented all the same analyses using random effects models - **in the supplementary tables** and described these in the text, particulalry where we made comparisons of the Cascade in different settings (for example pooled results in cohorts with and without LTBI testing). The cumulative proportions were higher in most analyses when compared to the fixed effect analysis, and of course the confidence intervals were wider. But the relative comparisons were unchanged. Hence several of the important messages of the paper were unchanged, such as comparing Cascade losses between lower income vs. higher income settings, or settings using or not using LTBI tests.

24-Pages 10 to 12 I realise there are many analyses summarised here, but I still think we need more detail. Since the authors already have the data stored it should be possible to generate forest plots which could go into the supplementary material so that we can see how variable the studies were and whether there are any outliers or influential studies.

R: We agree with the reviewer. We generated forest plots for each of the proportions of the cascade-of-care, stratified by studies that did not use LTBI tests and use LTBI tests (Fig. S2 and Fig. S3) added explanation in methods (lines 186-187) and in results (lines 270-272)

25-Page 13 For the stratified analyses why not perform a formal test for whether the strata differ?

R: We agree, and have performed a formal meta-regression to test whether the factors on which we stratified (use of LTBI tests, or country level income) were significantly associated with cascade losses. This is now described in the methods (Lines 194-206), and results are presented (Lines 293-295).

26-Figure 1bI think there are a couple of typos here: Proportion 3 and Proportion 4 texts look to have been truncated.

R: Thank you for pointing that out. Indeed, when we transformed figure 1b in TIFF format, proportion 3 and proportion 4 were truncated. We did not see this and apologize for not noticing this mistake; we have corrected it.

Reviewer #2:

 Overall summary of critique

Bastos et al. present a well conducted systematic review and meta-analysis evaluating the cascade of latent TB (LTBI) care in PLHIV. This is providing important data to quantify the gaps in care with additional nuance regarding the outcomes with and without the use of LTBI tests. My main points of critique are that the authors should provide more information regarding how they collected data to include in the narrative review (as this could be a separate topic) and about the framing of the conclusion as emphasizing that the outcomes for PLHIV were better than for other high-risk populations because these outcomes remain below target and this risks an implicit judgement that detracts from the need to emphasize the need to close these gaps in this high risk population as well as others. The discussion should also include some of the nuances regarding the imperfect sensitivity of current LTBI testing in PLHIV and limited knowledge re: ART drug interactions with newer shorter regimens.

 Reviewer comments by section

 Abstract

27-Line 55-56 - I think this second conclusion sentence should be split in two as think you are saying the use of LTBI testing did not reduce the proportion of eligible PLHIV who completed TPT?

R: We agreed with the reviewer, and we rephrased the conclusion as: “*The cumulative proportion of PLHIV completing TPT was higher than was previously reported among other at-risk populations. Completion of TPT was similar in high and low-middle countries. The use of LTBI testing did not reduce the proportion of eligible PLHIV who completed TPT. However substantial losses remain in the cascade of care and continued efforts are needed to improve it.*”

Introduction

28-Line 77 - please clarify 'never received it' - does this mean did not receive a course of TPT or did not actually initiate TPT?

R: This statement came from WHO Tuberculosis Report 2020, which states that the coverage globally of TPT among PLHIV was 50% in 2019 (page 118. Table 6.1 on page 119). This refers to **50% never initiated**. We have clarified this in the text.

Methods

29-Lines 147-148: narrative synthesis methodology requires further information. Currently this appears to be a throwaway comment, but it is important to understand how the authors approached data collection to answer this question.

R: We agreed, and we revised the methods of the narrative synthesis (lines 206-211)

Results

30-Line 160 - were TST thresholds consistent i.e., always <5mm and were IGRA results always classified as positive only according to the manufacturer's recommendations?

R: We defined the tests as positive as they were defined in the included papers. We added this information in methods (lines 147-148)

31-Line 180 - re: main loss being provider recommendation for TPT- did studies always actually measure this/refer to this in the same way? Where does the 31.1% come from (do not see it in Table 2)?

R: Thank you for your comment. We have clarified in the text the percentage 31.1% (now after inclusion of more cohorts=33.8%). Briefly it was the result of the subtraction of 100%-66.2% (pooled estimate of proportion 3). The definition of provider recommendation was not the same in all studies. Some studies explicitly termed this step as “providers recommendation”, but other studies reported in their Methods section the eligibility criteria for patients to receive TPT. In these studies we assumed that the patients that met the centre’s eligibility criteria, had received a provider recommendation for TPT. We have clarified this in the methods section.

32-Lines 197-199 - please review phrasing here re: clarity around despite where the losses occurred?

R: Thank you for your comment, we have revised the sentence (lines 282-284)

Discussion

33-Line 222- would include HIV in first sentence and currently it is a bit vague - do you mean better than the overall rate observed as per the Alsdurf review or for the subgroup with HIV? I think this refers to the former but see comment re: overall conclusion. I think framing should be although considerably better than for other high risk groups, analysis of the LTBI cascade of care for PLHIV reveals large gaps at multiple stages. This is important because there is generally greater consensus and support for TPT in PLHIV and despite this the outcomes do not reach targets.

R: Thank you for pointing this out. Yes, we referred to the overall rate estimated by Alsdurf et al. We have revised this statement.

34-Lines 227- suggest more discussion of the study findings prior to discussing limitations i.e. would bring other paragraphs (from line 249 onwards) earlier

R: We re-organized the paragraphs of the discussion, into summary of findings, implications, limitations, and strengths.

35-Lines 224-226- while these findings are important, should clarify that this was not the primary purpose of the review (or was this in the protocol)? Would suggest having some additional context by first citing data from your quantitative findings first to highlight major gaps being TPT being recommended and then completed.

R: Although the primary objective of this review was the quantitative synthesis of the cascade-of-care, in the original protocol (submitted in PROSPERO) we included enablers and barriers of the cascade of care as a secondary research question. Hence we have kept the findings related to enabler/barriers, but placed these results towards the end, after the primary results, as suggested.

36-Line 242- again here you are comparing PLHIV to the entire population offered TPT in the earlier review so language should be clear about this as PLHIV are more likely to have additional support services/counseling re: importance of TPT (as you specify in the next sentence) so important to clarify that you are not referring to a comparison between the same populations.

 R: We agree with the reviewer and clarified the sentence.

37-Lines 262-267 - need to acknowledge the limitations (imperfect sensitivity) of these tests in PLHIV given reliance on immune response.

R: We added a sentence in discussion (line 331-332).

38-Lines 268-270 - this contradicts what you say in the abstract re: use of LTBI testing did not reduce proportion of people who completed TPT?

R: Thank you, we revised this to: “TPT may provide some benefit in high TB incidence settings if all PLHIV are treated without use of LTBI testing [87,88]. However, use of LTBI tests can identify those most likely to benefit (Campbell), and treatment without prior LTBI testing might expose PLHIV to a nontrivial risk of adverse events[89,90]. Furthermore, the health care expenditures for drugs, follow-up visits and tests including those related to AE, to provide TPT to PLHIV who may not benefit from this could be redirected to strengthening the LTBI cascade-of-care in those (with positive LTBI tests) who will benefit more from TPT”

39-Lines 279-284- need to comment on ART drug interactions that may limit use of rifamycins and highlight that clinician training is needed re: safety of shorter regimens with certain ART regimens

R: We agreed with the reviewer that it is important to mention that some ART regimens can have important potential interactions with rifamycins, especially the protease inhibitors. We added this in discussion (lines 354-358)

 40-Lines 286-287 - Is this really the conclusion the authors want to draw? Average 50% treatment initiation is still not high and below treatment targets. Suggest instead that conclusion should be along the lines of 'TPT initiation and completion in PLHIV fall short of global targets but are higher than observed for other high-risk populations'? We need to be more ambitious as a TB community so drawing the comparison to other data that demonstrates massive unacceptable gaps does not help to drive our ambition to do better.

R: In our original conclusion, we have mentioned that the 50% treatment initiation is much lower than other targets in PLHIV (90-90-90). However, in response to this comment we have modified this sentence to add the term: “relative success”.

41-Table 1. -Replace IDU with PWID –

R: Thank you, we have replaced this term.

Table 4

43-Re: staff training (enabler) and counseling as time consuming (barrier) - to confirm, this was for TPT initiation but did not pertain to TPT recommendation?

R: The studies that reported training as an enabler, and time consuming as a barrier, linked these to TPT initiation.

Reviewer #3:

Thank you for the opportunity to read this submission.

44-This piece was a remarkable compilation of data.  The analysis of bias was remarkable for the effort but not clear how it eliminated or improved your ability to improve a programs impact.

R: The analysis of potential bias is a basic element of all systematic reviews. In the studies included in this review we found the majority had potential issues with selection of study cohort members, making inferences more limited, and overall reducing the quality of the evidence. This is mentioned (also in Response to Reviewer 4) in the Discussion - Lines 367-370.

45- The inability to understand who was excluded in the studies dilutes your ability to understand the external validity of your findings.

R: We are not sure we understand this comment. Indeed, we were not able to gather information on the reasons that each individual person was lost, but we do not see how this would jeopardize the generalizability of our findings. We included a large number of cohorts (N=71), including cohorts from all WHO regions, from high, middle- and low-income settings, from centres that used or did not use LTBI tests, and different types of service where PLHIV were followed (HIV care vs. other care). This variation in setting should enhance the external validity of our findings. Among the excluded cohorts, most were excluded because they reported very small populations, which might have been too highly selected (so not generalizable), or simply did not report even two steps of the cascade-of-care, so could not be analyzed.

46- The "care cascade" has its greatest utility at the individual level (single patient),  at the clinic site  or system level where you can take the findings of your cascade and identify where you need to strengthen your system, i.e, identification, entry and retention.  If it is not applied to specific sites it has limited utility as a management tool to direct resources to problems that are contributing to your outcome.  As you move into provincial and national data aggregation you dilute the ability to apply corrective interventions along the cascade because your data is aggregated.  With aggregated data the ability to locate the barriers to ID, Testing, Initiation TPT, Completion of TPT etc, are precluded and remain disarticulated facts with no place to point the corrective action, to strengthen the cascade.

We agree with the reviewer that a local cascade of care analysis can provide useful information to fix problems locally. However, the purpose of this cascade-of-care meta-analysis is to highlight the common challenges that many LTBI programs are facing, such as the steps in care where losses occur more often. Our stratified analyses-comparing programs that used vs did not use LTBI tests showed that the use of LTBI tests were not a major barrier, even in low-income settings. The stratified analyses comparing high income to low- and middle-income settings helps make the case that provision of TPT can be very successful in resource poor settings. As well, the synthesis of barriers and enablers of the cascade-of-care can provide useful information on problems and solutions that might be helpful in other settings. We believe this information will be useful for policy makers and program managers to understand how well LTBI programs can work, where they may fail, and be able to apply lessons learned elsewhere to their specific setting.

47-the Rif vs INH discussion seemed disconnected from data presented and conclusions.

R: Two important, and we believe connected, findings in our review were that very few cohorts used short rifamycin regimens and that completion of TPT was associated with major losses. However, we agree that the connection between these findings was not clear, so in response to this comment, and also reviewer 2 and 4, we have revised the Discussion to more closely connect the findings of the study Lines 351-359).

Reviewer #4:

In this article the author evaluates the proportion of people living with HIV completing the tuberculosis preventive therapy, assessing the cascade of care, as well as the use of tests to detect the presence of latent tuberculosis infection as part of the cascade and compared similarities in high and low-middle income countries, identifying barriers and facilitators for retention.

This review to be the first specifically looking at the cascade of TPT in PLHIV, and the use of the network meta-analysis methodology is also novel, as prior reviews have conducted more traditional meta-analyses. A major advantage of the network meta-analysis is that this methodology exploits all available direct and indirect evidence. It yields more precise estimates of the intervention effects in comparison with a single direct or indirect estimate and can provide information for comparisons between pairs of interventions that have not been compared directly within an individual randomized trial (indirect comparisons). This method uses the intervention effects from each group of randomized trials and therefore preserves within-trial randomization. Also, the simultaneous comparison of all interventions of interest in the same analysis enables the estimation of their relative ranking for a given outcome. As such, this is an important strength of the manuscript. The manuscript is very well organized and clearly written.

R: Thank you for your comments.

A few points need revision:

 48-    In abstract, line 54, conclusion: Please rephrase for clarity: "Completion was similar in high and low-middle countries, and in low-middle income countries and the use of LTBI testing did not reduce the proportion of eligible PLHIV who completed TPT."

R: Thank you, we have revised the sentence as also suggested by reviewer 1.

49-    In order to most properly follow the PRISMA statement, the objective of the systematic review needs further revision, to include the cascade of care participants, interventions, comparisons, and specific outcomes of interest used to identify the articles of interest.

R: We agree with the reviewer and have clarified the objectives (lines 116-119)

50-    In Results, page 160, the sentence "either IGRA or TST (IGRA or TST)" is redundant, please check.

R: Thank you for pointing that out. We have corrected the redundancy.

51-    Despite results reported in this meta-analysis that by design is restricted to PLHIV are better than those reported for the broader population, it is very important to highlight these results in the context of a still fragmented TB-treatment and prevention service provision, which certainly negatively impact these results, despite the fact that the authors cite that PLHIV are already linked to care.

R: Thank you. We have added this point in our discussion (lines 323-326).

52-    In Table 2, there is a typo ("cohor") that needs correction.

R: Thank you for pointing that out. We have corrected the typo.

53-In the Discussion, please consider including the strength of evidence for each recommendation or outcome.

R: We have not formally made any recommendations, so formal rating of the strength of evidence seems unnecessary. However, since all studies were observational, and, as mentioned in Response to Reviewer 3, the majority had potential design issues with cohort member selection, we consider this low quality evidence. This is now mentioned in the Discussion (Lines 367-370)

54-    In the discussion, the authors mention that an important finding in this review was that the use of LTBI tests was not a barrier to TPT initiation or completion among cohorts that used them, and that  TPT recommendation and initiation was higher among cohorts that used LTBI tests, and suggest that the use of LTBI tests should be encouraged, not only in high income country where it is already part of care, but also in low and middle-income countries, where this review found numerous reports of its successful use. They also mention that LTBI treatment without prior LTBI testing will expose a substantial proportion of PLHIV to a nontrivial risk of adverse events without evidence of the benefit of TPT. These points need to be put in context considering that published manuscripts not necessarily translate programmatic data. Therefore, taking into account the great utility this review will have from a public health perspective, it is recommended the review of this statement, not to bring the impression that we are currently following guidance that may be harmful to patients, what isn't at all the case.

R: We agreed with the reviewer, and we have modified our comments about use and non-use of LTBI tests prior to TPT. (Lines 339-340)

55-    I suggest to include a paragraph on the most recent trials with shortened course LTBI which will most probably impact the later stages of the cascade, with higher % of finishing the prophylaxis course, although the earlier cascade steps are under the same constrains.

R: Thank you for pointing that out, we have expanded the paragraph where we discussed the short rifamycin regimens (lines 351-359).
