# Supplementary material for: The latent tuberculosis cascade-of-care among people living with HIV: A systematic review and meta-analysis
Source: PLoS Med. 2021 Sep 7;18(9):e1003703. doi: 10.1371/journal.pmed.1003703 (PMC8439450; doi:10.1371/journal.pmed.1003703)
Supplement: Attachment — Submitted filename: Cascade TPTinHIV ResponsetoReviewers_R2.docx [file pmed.1003703.s023.docx]

Montreal, 11 June 2021

REF: “The Latent Tuberculosis Cascade-of-Care Among People Living with HIV: A Systematic Review and Meta-Analysis" (PMEDICINE-D-20-06012R2)”

Dear Dr. Richard Turner,

Many thanks for your letter of June 11th, 2021, regarding the above titled manuscript.

We have revised our manuscript, based on your instructions, and the reviewers’ comments, and have also prepared a point-by-point response – (in blue below). We also submitted the marked-up text - in case needed.

We hope the revised manuscript is now acceptable for publication in PLOS Medicine, and look forward to hearing from you.

Yours very truly,

Dick Menzies and Mayara Bastos,

On behalf of all authors

**______________________________________________________________________________**

Requests from Editors:

Please resubmit your paper as a research article.

R: Done.

Please quote the date(s) of the new search in the abstract.

To the abstract, we suggest adding a sentence, say, quoting the proportion of participants in low- and middle-income countries, and broken down by WHO region.

R: Thank you, we included the requested information.

We also suggest adding a sentence to summarize participants' age, sex, and CD4 and ART status.

R: Thank you, we included the requested information.

In the abstract and throughout the text, please quote p values alongside 95% CI, where available.

R: We respectfully disagree with this request. We are presenting pooled proportions with their 95% CI for the different steps of the cascade-of-care and do not feel adding p-values would help the readers in the interpretation of our results, compared to the 95% CI. In table S11, where the odds ratios of the meta-regression are presented, we have given the 95% CI and p-values.

Please remove the information about study funding from the abstract. In the event of publication, this information will appear in the article metadata, via entries in the submission form.

R: Done.

At lines 80/81, we suggest quoting both numbers to one decimal place.

R: Done.

At line 221, please make that "1.8 million".

R: Done.

Please use the style "low- and middle-income" throughout.

R: Done.

Please use the journal name abbreviation "PLoS ONE" consistently in the reference list.

R: Done.

Are references 14 and 57-65 missing journal names?

R: We corrected the above references.

Please rename the attached PRISMA checklist "S1_PRISMA_Checklist" or similar and refer to it by this label at line 116.

R: Done.

Comments from Reviewers:

Reviewer #2:

PMEDICINE-D-20-06012_R2

The authors have responded thoughtfully and appropriately to the critiques I and the other reviewers raised and accordingly present a strengthened manuscript, which includes an updated search and additional studies as well as revisions that have improved the clarity and messaging of the manuscript. I thus do not have major recommendations to offer at this stage. I would however suggest that the authors consider adding text in the abstract and author summary conclusions that note that TPT recommendation and initiation was higher among cohorts that used LTBI tests. For example, they could add something to the effect of the following clause (in bold) in the author's conclusion: 'Although TPT recommendation and initiation was higher among cohorts that used LTBI tests, the use of LTBI testing did not reduce the proportion of eligible PLHIV who completed TPT.' The rationale for this suggestion is that this paper may have important policy implications and it is otherwise possible that it could be interpreted that LTBI testing should not be scaled up in LMICs, which is not in accordance with the author's suggested recommendations. Otherwise, the manuscript needs a careful review by the authors to correct a few minor typos but overall represents an important addition to the literature on this topic.

R: Thank you for your suggestion. We agree with the reviewer, and we changed the conclusion of the abstract and added a comment about this as a new bullet point in the author summary.
